# Genetic diversity of microsymbionts nodulating *Trifolium pratense* in subpolar and temperate climate regions

**DOI:** 10.1038/s41598-022-16410-0

**Published:** 2022-07-15

**Authors:** Marta Kozieł, Michał Kalita, Monika Janczarek

**Affiliations:** 1 Department of Industrial and Environmental Microbiology, Faculty of Biology and Biotechnology, Institute of Biological Sciences, Maria Curie-Skłodowska University, 19 Akademicka, 20-033 Lublin, Poland; 2 Department of Genetics and Microbiology, Faculty of Biology and Biotechnology, Institute of Biological Sciences, Maria Curie-Skłodowska University, 19 Akademicka, 20-033 Lublin, Poland

**Keywords:** Microbiology, Environmental sciences

## Abstract

Rhizobia are soil-borne bacteria forming symbiotic associations with legumes and fixing atmospheric dinitrogen. The nitrogen-fixation potential depends on the type of host plants and microsymbionts as well as environmental factors that affect the distribution of rhizobia. In this study, we compared genetic diversity of bacteria isolated from root nodules of *Trifolium pratense* grown in two geographical regions (Tromsø, Norway and Lublin, Poland) located in distinct climatic (subpolar and temperate) zones. To characterize these isolates genetically, three PCR-based techniques (ERIC, BOX, and RFLP of the 16S-23S rRNA intergenic spacer), 16S rRNA sequencing, and multi-locus sequence analysis of chromosomal house-keeping genes (*atpD*, *recA, rpoB, gyrB,* and *glnII*) were done. Our results indicate that a great majority of the isolates are *T. pratense* microsymbionts belonging to *Rhizobium leguminosarum* sv. *trifolii.* A high diversity among these strains was detected. However, a lower diversity within the population derived from the subpolar region in comparison to that of the temperate region was found. Multi-locus sequence analysis showed that a majority of the strains formed distinct clusters characteristic for the individual climatic regions. The subpolar strains belonged to two (A and B) and the temperate strains to three *R. leguminosarum* genospecies (B, E, and K), respectively.

## Introduction

Fabaceae (Leguminosae) is the third largest plant family in the world. It includes about 19 500 herb, shrub, vine, and tree species within 770 genera occurring mainly in terrestrial habitats^1–4^. These leguminous plants are valuable protein sources for animal feed and human diet. Furthermore, they have an important role in crop rotation and are used for production of wood, tannins, oils, dyes, and medicines and in the horticultural trade^1,5^. Most species of Fabaceae plants can establish nitrogen-fixing symbioses with soil bacteria, collectively known as rhizobia^6,7^. This process, called biological nitrogen fixation (BNF), is an ecological and low-cost alternative providing nitrogen to legume crops. BNF decreases the amounts of synthetic nitrogen fertilizers applied in agriculture, and thus limits its adverse impacts on natural ecosystems (e.g., it reduces greenhouse gas emissions and pollutions of surface and underground waters)^8–10^. This process yields about 122 million tons of fixed nitrogen per year into the environment, with 50–70 million tons of N_2-_ fixed biologically by agricultural crops^9–11^. The establishment of an effective symbiosis involves a coordinated exchange of several signals of both plant and bacterial origins (i.e., plant flavonoids, rhizobial Nod factors, and exopolysaccharides)^12,13^. This “molecular dialogue” leads to formation of special new organs on host plant roots, called nodules, inside which rhizobia differentiate into bacteroides reducing atmospheric dinitrogen (N_2_) into ammonia, which is then used by the host plant^6,10,14,15^. These symbiotic interactions are host specific; this means that a given rhizobial species associates with a specific range of host legumes. Numerous studies have shown that some rhizobial species nodulate many host plants. For example, *R. leguminosarum* sv. *viciae* can infect *Vicia*, *Pisum*, *Lens*, and *Lathyrus; R. gallicum* can infect legumes from the genera *Phaseolus, Sesbania, Caliandra, Gliricidia*, and *Piptadenia*^16–20^; while other rhizobia have a very narrow host plant range. For example, *R. leguminosarum* sv. *trifolii* can establish symbiosis only with plants from the genus *Trifolium* (clovers), e.g., *T. pratense, T. repens*, and *T. rubens*^21–27^. Rhizobia are characterized by large and complex genomes (6–9 Mbp), which consist of a chromosome along or with several large plasmids (from one to six) ranging in a size from ca. 100 kb up to 2 Mb^28–30^. These bacteria are able to exist in three forms: as free-living organisms in the soil, as endophytes in various plants, or as endosymbionts inside legume root nodules^31,32^.

*Trifolium* spp. is one of the most important genera of the Fabaceae family, with more than 255 species spread across the world. These plants occur particularly frequently in the temperate and sub-tropical regions of North and South America, Europe, and Africa^33,34^. However, some *Trifolium* spp. are also found in subpolar regions^35–37^. Among them, the red clover (*Trifolium pratense* L.) is one of the most cultivated forage plants in Europe. Clover roots are nodulated by *R. leguminosarum* sv. *trifolii* strains. Several studies have indicated the occurrence of *R. leguminosarum* sv. *trifolii* strains in different (temperate, subtropical, arctic, and subarctic) zones^38–40^. As shown by other researchers, the differences in the genetic structure and composition of rhizobial populations might be associated with geographical distance and local environmental conditions in the region^41,42^. In the absence of compatible host plants, rhizobia must often survive long periods as saprophytes in the soil. In such periods, rhizobia are exposed to the action of several abiotic stress factors, such as soil pH, salinity, drought, variable temperatures, and heavy metals, which can affect the genetic composition of their populations^10,43,44^.

To give more insight into the influence of some environmental factors, such as low temperature, on the genetic diversity of *T. pratense* microsymbionts, rhizobial strains were isolated from root nodules of red clover plants grown in two European regions, essentially differing in annual temperature profiles: the subpolar climate zone (Norway, Tromsø region, 69°38′36-40″ N, 18°54′00-01″ E) and the temperate climate zone (Poland, Lublin region, 51°15′55-57″ N, 22°32′6-10″ E), and analyzed in this study. The Tromsø region is located in the north of the Arctic Circle, where the vegetation session is very short (2–3 months) and the average temperature of the hottest month (July) is only 12 °C. In contrast, the vegetation season in the Lublin region is almost twice longer and the average temperature in July is nearly two times higher (20 °C). To establish and compare the genetic diversity of the *T. pratense* microsymbionts derived from these different temperature zones, several genetic analyses, i.e., genomic DNA fingerprinting using Enterobacterial repetitive intergenic consensus (ERIC-PCR), BOX-PCR, and restriction fragment length polymorphism of the 16S-23S intergenic transcribed spacer (ITS PCR–RFLP) were carried out. Moreover, to determine the genomic relationships between the studied isolates, sequence analyses of five house-keeping genes as individuals and Multi-Locus Sequence Analysis (MLSA) using their concatenated sequences for representatives of both strain populations were performed.

## Results

### Identification of red clover microsymbionts using 16S rDNA sequence analysis

To establish whether low temperature influences the genetic diversity of *T. pratense* root nodule microsymbionts, a comparative genetic analysis of strains derived from the two geographical regions located in different climatic (temperate and subpolar) zones, which differ essentially in respect to temperature conditions, was performed. For this approach, bacteria occupying root nodules of red clover plants grown in these two climatic zones were isolated. In total, 120 strains were obtained and further analyzed (60 strains for each climatic zone). The native rhizobial isolates were Gram-negative, fast-growing bacteria, which formed single colonies with diameters of 2–4 mm, white or creamy, mucous, raised, and circular with entire margins.

Firstly, to determine to which rhizobial species these red clover isolates show the highest sequence similarity, total genomic DNA from these bacteria was isolated and the analysis of the 16S rRNA gene was performed. We established that almost all of the tested strains (115 from 120 strains, i.e. 95.83%) isolated from root nodules of the red clover plants from these two climatic zones showed high 16S rRNA sequence identity to homologous genes of strains belonging to *R. leguminosarum* species (sequence identity 99–100%). Only 5 strains derived from the subpolar region (i.e., R14, R52, R104, R123, and R125) exhibited significantly higher sequence identity to *Pararhizobium giardinii* (from 97.89 to 100%) than to *R. leguminosarum* species. Next, to check whether the 120 strains are able to establish nitrogen-fixing symbiosis with *T. pratense*, glass tube experiments with the use of nitrogen-free medium and red clover seedlings were conducted. We confirmed that all strains identified as *R. leguminosaum* using the 16S rRNA sequence analysis were able to re-nodulate this host, indicating that they are true microsymbionts of red clover plants. They induced formation of pink nodules on the roots, indicating that these nodules are effective in nitrogen fixation (Nod + Fix +). The only exceptions were the R14, R52, R104, R123, and R125 strains, which did not induce nodules on the *T. pratense* roots (Nod-), confirming that they are not microsymbionts of this legume. In conclusion, our results indicate that a great majority of strains isolated from the root nodules of red clover plants grown in the temperate and subpolar climatic regions belong to one *R. leguminosaum* species.

### Determination of the genetic diversity and phylogenetic relatedness of red clover microsymbionts using PCR-based methods

Next, to compare the genetic diversity of *R. leguminosaum* strains derived from the two different climatic zones, we used three PCR-based techniques (ERIC-PCR, BOX-PCR, and PCR–RFLP of 16S-23S rRNA ITS). For this purpose, genomic DNA of the 115 strains and primers specific for repetitive consensus sequences were used. The amplicons obtained in the ERIC-PCR and BOX-PCR techniques were electrophoretically separated using 3% (w/v) agarose gels and analyzed in order to construct trees showing phylogenetic relationships between these rhizobial strains.

**Figure 1:**
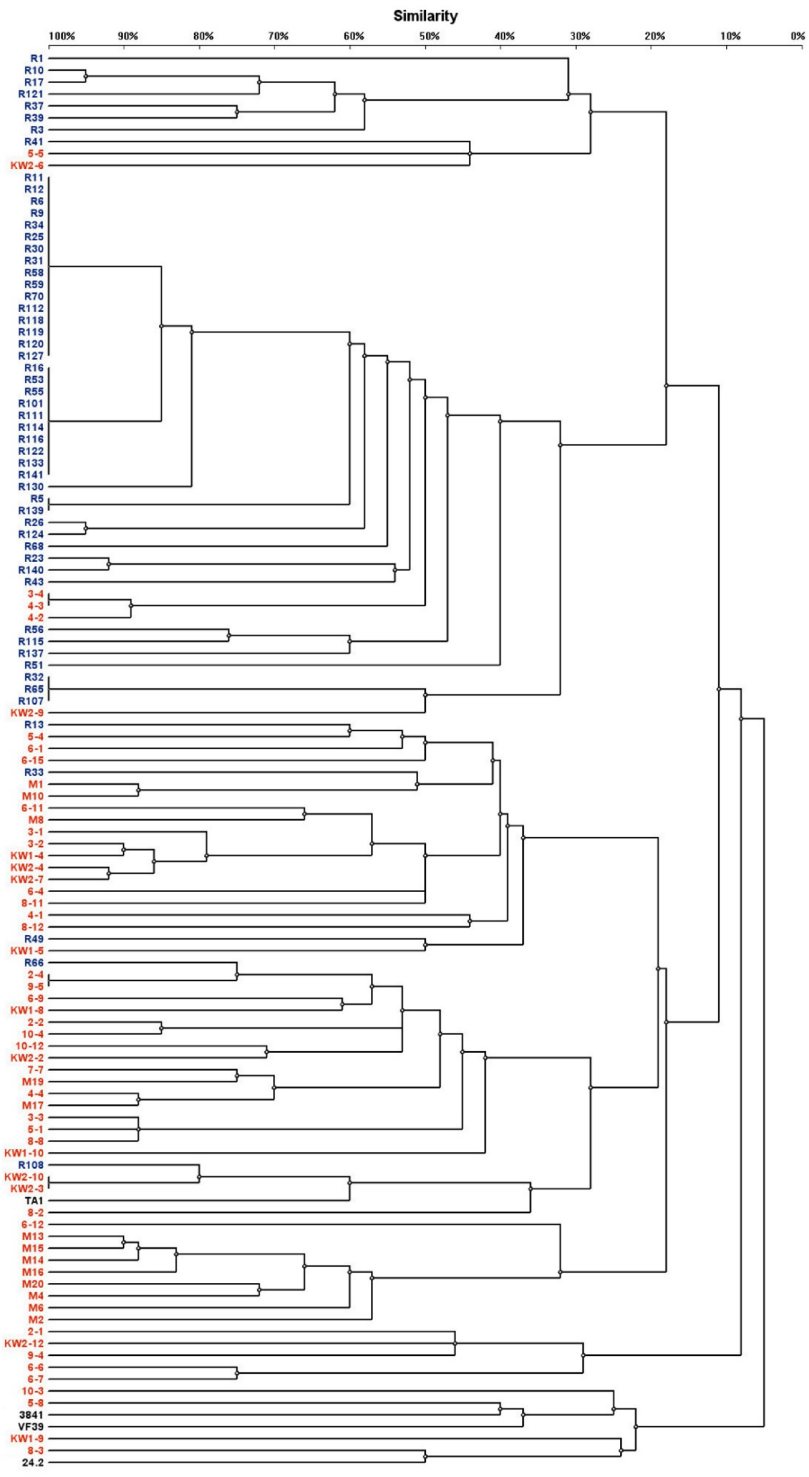
Dendrogram constructed on the basis of the ERIC-PCR analysis showing the genetic diversity of *R. leguminosaum* sv. *trifolii* strains originating from two different climatic zones. The dendrogram was performed using the Dice coefficient and the UPGMA method.

**Figure 2:**
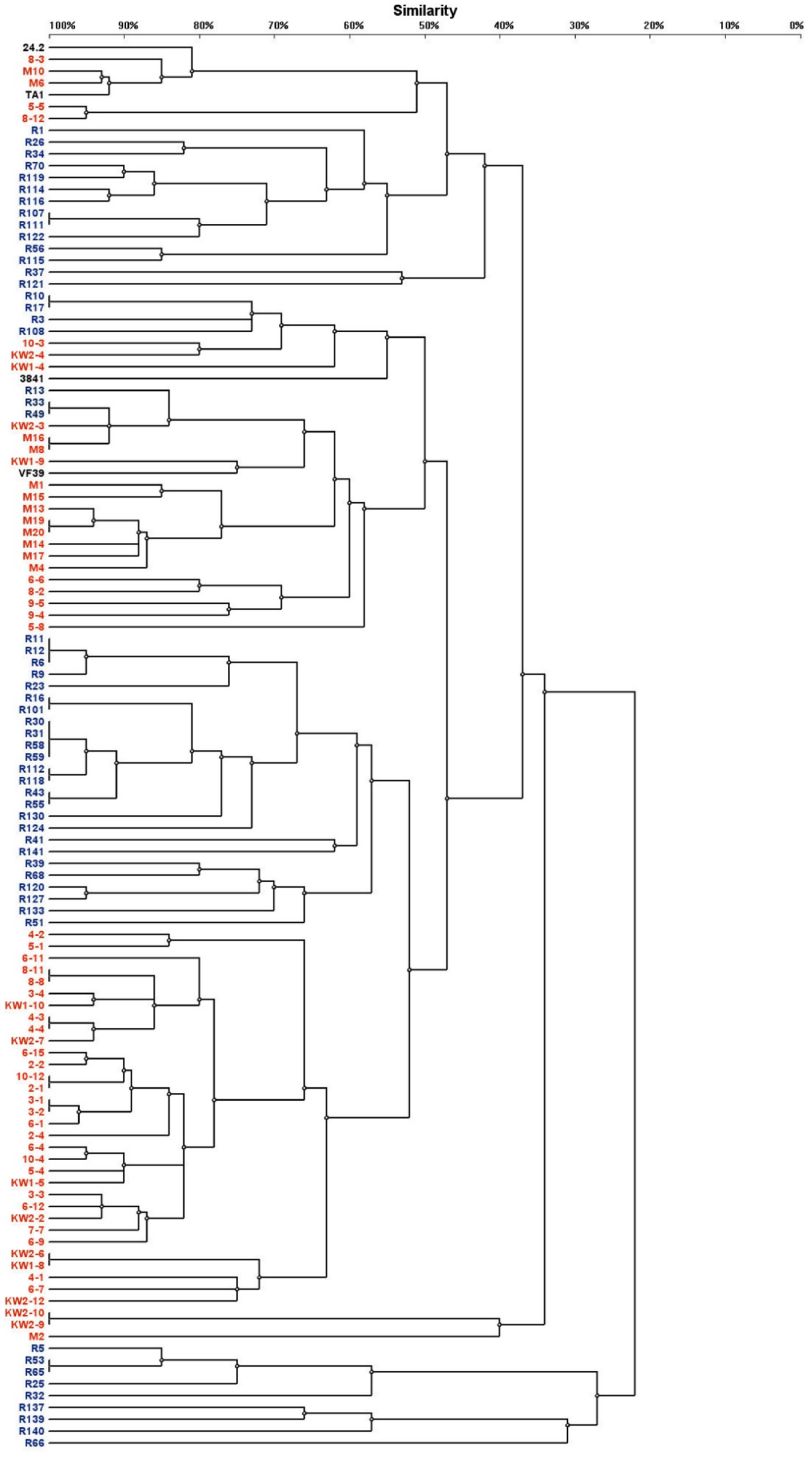
Dendrogram constructed on the basis of the BOX-PCR analysis showing the genetic diversity of *R. leguminosaum* sv. *trifolii* strains originating from two different climatic zones. The dendrogram was performed using the Dice coefficient and the UPGMA method.

In general, a slightly lower number of genomic patterns were found for the studied strains in ERIC-PCR than BOX-PCR, suggesting that the latter analysis is more informative and has higher discrimination power than the former (Figs. 1 and 2). In total, 85 fingerprint patterns were identified in the strains analyzed using ERIC-PCR: 57 patterns were found within the temperate population strains, whereas only 28 patterns were detected in the strains from the subpolar climate population (Fig. 1, Supplementary material Table S1). Among the identified ERIC-PCR patterns, a high diversity in respect to the number and size of PCR fragments was found; from 1 (strains KW1-9 and KW2-9) to 15 (strain 6-1) PCR fragments were identified, which had the length from 171 to 2905 bp (Table S1). A great majority of the patterns were unique and specific for individual strains, whereas only a few fingerprint patterns were characteristic for more than one strain. Unique fingerprint patterns were also detected for the four tested reference *R. leguminosarum* strains (TA1, 3841, 24.2, and VF39). The most frequently represented patterns were 4 and 7, which were identified in 16 and 10 strains of the subpolar origin, respectively. Thus, ERIC-PCR identified a higher number of genomic profiles within the temperate than subpolar strains, which suggests a higher genetic diversity in the temperate than subpolar populations.

The BOX-PCR analysis identified in total 96 genomic patterns, with 44 patterns within the subpolar climate population and 52 within the temperate climate population, respectively (Fig. 2, Table S2). As in ERIC-PCR, each of the tested reference *R. leguminosarum* strains (TA1, 3841, 24.2, and VF39) possessed a unique fingerprint pattern in this analysis.

Similarly to ERIC-PCR, BOX-PCR revealed a high diversity in respect to the number and size of PCR fragments, from 2 (strains KW2-9 and KW2-10) to 17 (strain R139) PCR fragments with the length from 260 to 4526 bp. Only two BOX-PCR patterns (4 and 12) were found in more than two strains (Table S2). Thus, a great majority of the patterns were unique and specific for only individual strains.

In conclusion, we found a slightly higher number of genetic patterns in the temperate strains than in the subpolar strains using BOX-PCR, which confirms a higher genetic diversity of the former population in comparison to the latter one. Moreover, our results indicate that BOX-PCR is a more discriminative technique for DNA fingerprinting of rhizobial strains than ERIC-PCR.

Based on the two analyses described above, we found that a majority of the studied isolates from the temperate zone grouped together in distinct clusters separated from those formed by the subpolar zone isolates (Figs. 1 and 2). Similarly, a majority of the subpolar origin strains formed tight groups. Interestingly, some strains from these two geographical regions were found in the same clusters, suggesting their close phylogenetic relatedness.

Next, we performed RFLP analysis of the 16S-23S rDNA ITS fragments obtained in PCR using primers FGPS1490 and FGPL132’ and three restriction enzymes. Restriction fragments obtained after digestion by *Bsu*RI, *Taq*I, and *Msp*I were separated by electrophoresis in 3% (w/v) agarose gels and analyzed. Patterns obtained in the digestion of the 16S-23S rRNA ITS by the individual enzyme were analyzed for each of the studied strains separately and subsequently together for all the three enzymes (Tables 1 and S3, Figs. S1–S3).

**Table 1:** 16S-23S ITS genotypes and PCR–RFLP groups obtained for *R. leguminosarum* sv. *trifolii* using enzymes *Bsu*RI, *Taq*I, and *Msp*I.

Strain	Restriction patterns of 16S-23S rDNA ITS after enzyme cutting*			ITS-PCR Genotype**	PCR–RFLP group***
	*Bsu*RI	*Msp*I	*Taq*I		
R1 R3 R5 R6 R9 R10 R11 R12 R17 R25 R30 R31 R37 R39 R58 R59 R65 R70 R101 R112 R115 R118 R124 R127 R130 R137	A	A	A	AAA	I
R133	A	A	D	AAD	II
R16 R23 R34 R43 R51 R53 R66 R68 R107 R114 R116 R119 R120 R122 R141	B	B	B	BBB	III
R41	C	C	C	CCC	IV
R13 R26 R32 R33 R49 R55 R56 R108 R111 R121 R139 R140 2-1 3-1 3-2 3-3 3-4 4-1 4-2 4-3 4-4 5-4 6-9 6-12 6-15 8-8 8-11 9-4 10-4 10-12 KW1-4 KW1-5 KW1-8 KW1-10 KW2-2 KW2-3 KW2-6 KW2-9 KW2-10 KW2-12 M1 M2 M4 M13 M14 M15 M16 M17 M19 TA1 Rlv3841 VF39	D	D	D	DDD	V
2-4 6-1 KW2-4 KW2-7 M8	D	G	D	DGD	VI
6-4 7-7 M20	E	E	E	EEE	VII
M6 M10	F	F	F	FFF	VIII
KW1-9	F	L	K	FLK	IX
2-2 10-3 Rt24.2	G	K	F	GKF	X
6-11 8-3	G	M	G	GMG	XI
5-8 9-5	H	H	H	HHH	XII
5-5 6-6	I	I	I	III	XIII
6-7	I	I	D	IID	XIV
5-1 8-2 8-12	J	J	J	JJJ	XV

*The letters indicate the types of restriction patterns of the 16S-23S rRNA ITS obtained after digestion with the individual restriction enzymes.

**Group letters indicate the RFLP 16S-23 rRNA genotypes of the tested strains resulting from the combined analysis of restriction fragments derived from digestion with three endonucleases.

***Roman numerals indicate the RFLP 16S-23S rRNA genomic groups of the tested strains resulting from the analysis of individual genotypes.

When *Bsu*RI was used, 10 RFLP patterns were found (named from A to J), in which 50–475 bp fragments were identified. The number of DNA fragments in these patterns ranged from 3 (pattern G) to 6 (patterns A, B, and D) (Table S3). A slightly higher diversity in RFLP of the 16S-23S rDNA ITS was found for *Msp*I digests, i.e., 13 patterns (named from A to M) were identified, in which from 4 (patterns I and L) to 7 (A-G, J, and K) fragments of the length from 40 to 435 bp were detected. In the case of the *Taq*I enzyme, 11 RFLP patterns (named from A to K) were found, in which 75–410 bp fragments were identified. The number of DNA fragments in these profiles ranged from 5 (C, F, G, H, J, K) to 6 (A, B, D, E, I). In conclusion, within the temperate climate population, a majority of the RFLP patterns were characteristic only for a low number of the strains or were even strain-specific. These data confirm the higher genetic diversity of the strains coming from the temperate zone in comparison to the strains of the subpolar origin.

Furthermore, based on the simultaneous analysis of all RFLP patterns of 16S-23S rDNA ITS using three restriction enzymes, 15 groups (I to XV) within the studied *R. leguminosarum* sv. *trifolii* strains were identified (Table 1). Among them, group V was the most abundant one (49 strains, 40.8%) containing strains from both populations: 12 from the subpolar zone and 37 from the temperate zone, respectively. Also, the reference *R. leguminosarum* TA1, Rlv3841, and VF39 strains were classified into this group. The other RFLP groups were significantly less frequent. Groups I and III included exclusively strains from the subpolar zone, comprising 26 strains (21.6%) and 15 strains (12.5%), respectively. The other groups were even smaller: group VI contained 5 strains (4.16%), groups VII, X, and XV had 3 strains each (2.5%), and groups VIII, XI, XII, and XIII comprised 2 strains each (1.66%). The other RFLP profiles were unique for individual strains (II-R133, IV-R41, IX-KW1-9, and XIV-6-7). Interestingly, the subpolar population strains were classified only to 4 RFLP patterns (I-IV) and to the large group V (including also the temperate zone strains).

In contrast, a higher number of PCR–RFLP pattern groups were detected in the case of the temperate zone strains (10 groups). Among them, groups VI-XV contained exclusively strains from the temperate zone, and group V included strains from both populations. In summary, 15 distinct genomic groups within the *R. leguminosarum* strains were found in the RFLP analysis of the 16S-23S rRNA ITS with enzymes *Bsu*RI, *Taq*I, and *Msp*I. A lower number of PCR–RFLP groups within the subpolar population isolates (5 groups) than within the temperate population isolates (10 groups) were found.

In conclusion, our results obtained using three PCR-based analyses (ERIC-PCR, BOX-PCR, and PCR–RFLP of the 16S-23S rRNA ITS) demonstrated a high genetic diversity of the strains isolated from root nodules of *T. pratense* plants. Moreover, the isolates from the temperate zone exhibited a higher genetic diversity than those from the subpolar zone. These data confirm that a low temperature is an important stress factor exerting a negative effect on bacterial survival in the environment, and in consequence, on the genetic diversity of *R. leguminosarum* sv. *trifolii* strains.

### Determination of genetic diversity and phylogenetic relatedness of red clover microsymbionts using MLSA

Next, representative isolates from both populations were characterized using phylogenetic analyses of five house-keeping genes (*atpD*, *rpoB*, *glnII*, *recA*, and *gyrB*). In total, 30 strains were chosen for MLSA (15 strains from each climate zone), which represented a high diversity of these two populations shown by the fingerprinting analyses. The temperate zone population was represented by KW1-9, KW2-9, 2-2, 3-1, 3-3, 4-3, 5-8, 6-11, 8-3, 8-11, 10-3, M2, M14, M16, and M19, whereas the subpolar zone population comprised R1, R13, R23, R26, R32, R41, R49, R51, R53, R56, R66, R70, R108, R118, and R137.

**Figure 3:**
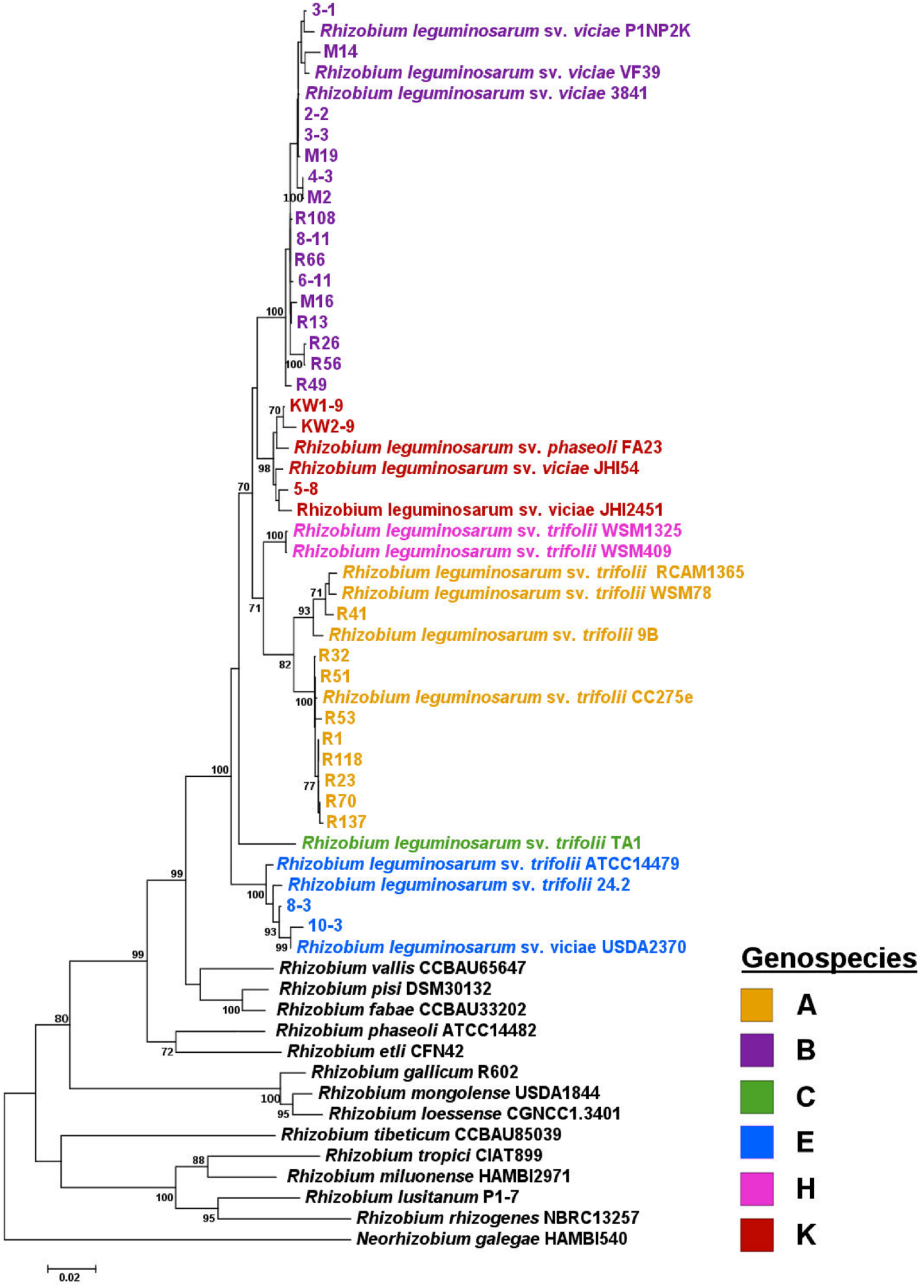
Maximum Likelihood tree based on concatenated sequences of five house-keeping genes (3054 bp) showing relationships of the representative red clover isolates with selected members of different *R. leguminosarum* genospecies and reference strains for *Rhizobium* species. The colors indicate genospecies. Bootstrap values (based on 1000 replicates) are shown on the branches. The scale bar represents the number of nucleotide substitutions per site. The phylogenetic analysis was conducted in MEGAX using the Maximum Likelihood algorithm with the General Time Reversible model plus Invariant site plus Gamma rate distribution (GTR + I + G).

In general, similar phylogenetic relatedness between the representative strains of the two *R. leguminosarum* sv. *trifolii* populations in trees constructed on the basis of genomic sequences of individual house-keeping genes as in the tree obtained using MLSA was found (Figs. 3, S4–S8). The phylogenetic trees were constructed using the Maximum-Likelihood (ML) method. For MLSA, a sequence with the total length of 3,054 bp was used, which contained partial sequences of *atpD* (432 bp), *recA* (495 bp), *rpoB* (855 bp), *gyrB* (654 bp), and *glnII* (618 bp). The phylogenetic analysis of each individual gene and concatenated chromosomal genes confirmed the taxonomic affiliation of the isolates. These newly isolated red clover strains grouped in several well-defined lineages, together with their closely related *R. leguminosarum* strains (e.g., 3841, WSM1325, and ATCC14479), with sequence identity values in the range of 92.1–100% for *atpD,* 95.9–99.7% for *recA,* 95.5–100% for *rpoB,* 95.2–100% for *gyrB,* and 93.3–100% for *glnII.* The bootstrap value at which all studied strains grouped with the reference *R. leguminosarum* strains was 100%.

Similarly, the ML phylogeny inferred based on the concatenated sequences of the five chromosomal loci clearly delineated the studied strains into confident clades, and they all exhibited the closest relatedness to *R. leguminosarum* species. In general, a high degree of heterogeneity was confirmed within the *R. leguminosarum* group, including also reference strains. The matrix data obtained for the concatenated sequences indicate heterogeneity of 94.9%–100% among the studied strains, 94.9–99.9% between the studied and reference strains, and 95.9–98.8% among the only reference strains. In the tree constructed on the basis of MLSA, two large clades were found, in which strains exclusively derived from one climatic zone were grouped (i.e., temperate zone strains: 2-2, 3-1, 3-3, 4-3, M2, M14, and M19 and subpolar climate strains: R1, R23, R32, R41, R51, R53, R70, R118, and R137) (Fig. 3). Moreover, two small groups comprising strains from the temperate zone (i.e., the first group encompassing KW1-9, KW2-9, and 5-8, and the second group encompassing 8-3 and 10-3) were found. These data indicate that strains from the same climatic region present closer phylogenetic distance. Interestingly, one large group of strains from both subpolar and temperate zones (6-11, 8-11, R13, R26, R56, R66, R108) was found, which was located between two large groups encompassing exclusively the strains from the temperate region. Our data suggest close phylogenetic relatedness between these strains despite their different geographical origins.

Based on a recent study of Young and colleagues^57^, who have provided evidence for the occurrence of multiple genospecies within the *R. leguminosarum* species complex (Rlc) and have shown that a few house-keeping gene sequences are sufficient to assign strains to appropriate genospecies, we classified the representatives of the two climate populations into four genospecies (A, B, K, and E) (Fig. 3). Sixteen of the 30 studied strains belonged to gsB (2-2, 3-1, 3-3, 4-3, 6-11, 8-11, M2, M14, M16, M19, R13, R26, R49, R56, R66, R108; 53.33%) similarly as the reference strain 3841 and a divergent member of gsB, P1NP2K. Strains from both climatic zones were present in this genospecies. Based on the matrix data, the heterogeneity among them was 98.4–100%, and 98.5–99.9% between the studied and reference strains (99.3–99.5% among the only reference strains). The number of strains assigned to gsA was lower (nine strains: R1, R23, R32, R41, R51, R53, R70, R118, R137; 30%). These strains derived only from the subpolar region and formed a clade together with reference strains CC275e, 9B, WSM78, and RCAM1365, which are members of this genospecies (the matrix data indicated 97.3%*–*100% heterogeneity among the studied strains, 97.4%–99.9% between the studied and reference strains, and 97.5–99.4% among the reference strains). Two small groups containing the temperate strains were classified to gsK (strains KW1-9, KW2-9, and 5-8; 10%) and gsE (strains 8-3 and 10-3; 6.67%), respectively, since they grouped together with the reference strains from these genospecies (gsK -FA23, JH54, JH2451 and gsE -USDA2370 and 24.2). Based on the study conducted by Young and colleagues^57^, gsE is the species *R. leguminosarum *sensu stricto, because it includes the type strain USDA2370. The matrix data for the gsK group indicated 98.5–99.4% heterogeneity among the studied strains, 98.5–99.2% between the studied and reference strains, and 98.9–99.1% among the reference strains. In the case of the gsE group, the heterogeneity between strains 8-3 and 10-3 was 98.9%, whereas the heterogeneity between the studied and reference strains was estimated at 98.5–99.4% and among the reference strains at 98.8–99.0%. In conclusion, the subpolar population representatives belonged to genospecies A (9 strains) and B (6 strains), whereas the temperate population representaives belonged to genospecies B (10 strains), K (3 strains), and E (2 strains), respectively. When the origin of these strains from individual clover plants was analyzed in details (Table 2), it was found that all plants from the subpolar region were infected by the strains from both genospecies and a ratio of gsA to gsB strains was ~ 1:1 (the only exception was plant 4). Namely, strains R1 (gsA) and R13 (gsB) were isolated from nodules of plant 1, strains R23, R32, R42 (gsA), R26, and R49 (gsB) from nodules of plant 2, strains R51, R53, R70 (gsA), R56, and R66 (gsB) from nodules of plant 3, R118 (gsA) from a nodule of plant 4, and strains R137 (gsA), and R108 (gsB) from nodules of plant 5, respectively. These results suggested similar frequency of occurrence of both genospecies not only on the population level, but also in individual subpolar plants tested. Interestingly, a higher diversity among the temperate plants was found in respect to both a number of representative strains coming from individual plants and their genospecies. Namely, strains 2-2, 3-1, 3-3, 4-3 (gsB), and 5-8 (gsK) derived from nodules of plant 1, strains 6-11, 8-11 (gsB), 8-3, and 10-3 (gsE) from nodules of plant 2, KW1-9 (gsK) from a nodule of plant 3, KW2-9 (gsK) from a nodule of plant 4, and strains M2, M14, M16, and M19 (gsB) from nodules of plant 5, respectively. Thus, gsB was dominant, whereas gsA was absent within the temperate zone representatives. Moreover, it is worth noting that the strains assigned to gsK were isolated from different clover plants (nos. 1, 3, and 4).

**Table 2:** Bacterial strains used in this study.

Strains	Characteristics	Source
R1, R3, R5, R6, R9, R10, R11, R12, R13, R16, R17 (plant 1); R23, R25, R26, R30, R31, R32, R33, R34, R37, R39, R41, R43, R49 (plant 2); R51, R53, R56, R58, R59, R65, R66, R68, R70 (plant 3); R101, R107, R108, R111, R112, R114, R115, R116, R118, R119 (plant 4); R120, R121, R122, R124, R127, R130, R133, R137, R139, R140, R141 (plant 5)	Strains isolated from root nodules of five red clover plants grown in the subpolar climate region (Tromsø region, 69°38′ 36-40″ N, 18°54′00-01″ E)	This study
2-1, 2-2, 2-4, 3-1, 3-2, 3-3, 3-4, 4-1, 4-2, 4-3, 4-4, 5-1, 5-4, 5-5, 5-8 (plant 1); 6-1, 6-4, 6-6, 6-7, 6-9, 6-11, 6-12, 7-7, 8-2, 8-3, 8-11, 8-12, 9-4, 9-5, 10-3, 10-4, 10-12 (plant 2); KW1-4, KW1-5, KW1-8, KW1-9, KW1-10 (plant 3); KW2-2, KW2-3, KW2-4, KW2-6, KW2-7, KW2-9, KW2-10, KW2-12 (plant 4); M1, M2, M4, M6, M8, M10, M13, M14, M15, M16, M17, M19, M20 (plant 5)	Strains isolated from root nodules of five red clover plants grown in the temperate climate region (Lublin region, 51°15′ 55-57″ N, 22°32′6-10″ E)	This study
*R. leguminosarum* sv. *trifolii* 24.2	Spontaneous rifampicin-resistant mutant of isolate Rt24 obtained from *T. pratense* root nodules	^76^
*R. leguminosarum* sv. *trifolii* TA1	Strain isolated from *T. subterraneum* root nodules	^77^
*R. leguminosarum* sv. *viciae* 3841	Spontaneous streptomycin-resistant mutant of isolate 300 obtained from *Pisum sativum* root nodules	^28^
*R. leguminosarum* sv. *viciae* VF39	Strain isolated from *Vicia faba* root nodules	^78^

Next, when the MLSA tree and those constructed on the basis of the individual house-keeping genes were compared, high consistency between them was found (Figs. 3 and S4–S8). A great majority of the strains in the individual gene trees clustered in similar distinct groups, including the same or very similar sets of strains as in the MLSA tree. In the case of the *gyrB* tree, the composition of strains in all groups and their classification into particular genospecies was identical as in the MLSA tree (i.e., 9 strains assigned to gsA, 16 to gsB, 3 to gsK, and 2 to gsE) (Figs. 3 and S6). Very similar results to those from MLSA were obtained using sequence analysis of the *atpD*, *recA*, and *rpoB* genes. The only exception was the presence of R49 in the *atpD* tree (Fig. S4) and the presence of R26 and R56 in the *recA* tree (Fig. S5) among the strains classified to gsA (not to gsB as in MLSA) as well as the presence of KW2-9 in the *rpoB* tree among the gsB strains (not within the gsK group as in MLSA) (Fig. S7). More differences in the strain composition of individual clusters in comparison to the MLSA tree were found in the *glnII* tree. Namely, three strains (M2, M19, and 4-3) located within the gsB group in all these trees grouped together with KW1-9, KW2-9, and the reference strains for gsK, whereas strain 5-8 was found among the gsB strains, instead of those belonging to gsK (Fig. S8). Interestingly, the highest consistency was found for strains 8-3 and 10-3, which were classified to gsE based on MLSA and all individual house-keeping gene analyses. Thus, a high similarity in the number of groups formed and their strain content were observed in the *atpD*, *rpoB*, *recA*, *glnII*, and *gyrB* trees in comparison to the MLSA tree constructed using the concatenated sequence of these genes.

In conclusion, we have shown the phylogeny of *R. leguminosarum* sv. *trifolii* strains from two geographical regions based on MLSA using *atpD, recA, rpoB, gyrB,* and *glnII* sequences. Based on this, a high degree of heterogeneity within the *R. leguminosarum s*trains was found. Using sequences of these genes from the reference strains belonging to different Rlc genospecies, the studied strains were assigned to four genospecies; among them, gsB and gsA were the most abundant, whereas gsK and gsE were significantly less frequent. Interestingly, a great majority of the strains from the individual geographical regions formed distinct groups (i.e., the subpolar strains assigned to gsA and the temperate strains assigned to gsK and gsE), whereas only few strains from these two populations formed a common group (assigned to gsB). These results confirm that MLSA is a rapid and reliable way of providing information on phylogenetic relationships of rhizobial strains and providing a possibility to classify strains into particular genospecies, in the case of bacterial species with a high genomic diversity, such as *R. leguminosarum*.

## Discussion

Rhizobia are an important group of soil bacteria due to their ability to establish nitrogen-fixing symbioses with many legume species, including those serving as essential sources of proteins in human and cattle diets as well as those used in crop rotation to increase nitrogen levels available to plants (e.g. cereal crops)^45^. Therefore, the use of rhizobia in sustainable agriculture reduces the need for synthetic nitrogen fertilizers. Among legumes, *Trifolium* (clovers) spp. are mainly distributed in the temperate zones of Europe, Asia, and America and they are among the most important fodder plants for animals^35,46^. It is known that bacteria belonging to *R. leguminosarum* sv. *trifolii* are effective microsymbionts of *Trifolium* spp. plants^21,35,47^.

Soil is a challenging environment for bacteria, in which conditions may change rapidly and bacteria have to acclimate and adapt in order to survive. The diversity of strains occupying nodules is a function of their biodiversity in the rhizosphere. To survive as saprophytes and to nodulate, rhizobia have to compete with other bacterial species and with other rhizobial strains, thus competitive traits are very important for nodulation success^21,48–51^. Therefore, studies on rhizobial biodiversity are an important approach in finding stress-tolerant native isolates^52,53^. As demonstrated in several papers, various environmental factors influence the composition and activity of rhizobial populations in soil and the rhizosphere. Among them, soil pH and temperature proved to be the major abiotic stress factors, which determine the diversity of the bacterial community^37,53–56^. Accordingly, the search for rhizobial isolates with high tolerance to stress conditions may be a way of improving legume yields, especially in more adverse climate and soil conditions. Apart from these studies, no comparative analyses of the genetic diversity of *R. leguminosarum* sv. *trifolii* strains isolated from clover plants grown in two distinct climatic zones with highly different temperature profiles have been done to date.

Therefore in this work, to broaden our knowledge of the influence of some environmental factors, such as a low temperature, on the genetic diversity of legume microsymbionts, we analyzed strains isolated from root nodules of *T. pratense* plants grown in two distinct climatic (subpolar and temperate) zones characterized by different annual temperature profiles (i.e., two European regions: Tromsø in Norway and Lublin in Poland). In total, 120 strains (60 strains derived from each region) were genetically characterized. Based on the 16S rRNA sequence analysis and re-nodulation plant tests, we indicated that nearly all of these strains (96%) effectively nodulated this host and were classified to symbiovar *trifolii* of the *R. leguminosarum* species. Our results are in congruence with other earlier data on *Trifolium* spp. microsymbionts^4,15,24–27,39,55,57^.

To compare the diversity and establish the genomic relationships between the *R. leguminosarum* strains from the two populations, DNA fingerprinting using three PCR-based techniques was performed (ERIC-PCR, BOX-PCR, and PCR–RFLP of 16S-23S ITS). These techniques are well-known for their discrimination power, since they generate highly specific and reproducible patterns that enable accurate strain differentiation^58^. Using these approaches, we found a high diversity within both populations of *R. leguminosarum* sv. *trifolii* strains (85 ERIC and 96 BOX patterns, respectively). Furthermore, a significantly higher genetic diversity of the strains from the temperate zone than those from the subpolar zone was found (it was especially noted in the ERIC-PCR and RFLP analyses). This suggests that a low temperature exerts a negative effect on both the genetic diversity and the structure of the strains associated with clover plants from the subpolar region. However, it cannot be excluded that other factors, such as geographical distance and local environmental conditions, may also influence the genetic diversity of these strains. Our results indicated that a great majority of the strains from the analyzed populations grouped in clusters characteristic for the geographical regions of their origin. Only a low number of the strains from both geographical regions resembled identical ERIC-PCR and/or BOX-PCR patterns. Similarly to our findings, other researchers observed that other environmental factors also influence the diversity and composition of rhizobial populations. For an example, Van Cauwenberghe and colleagues^44,59^ revealed that differences in the genetic composition of a population of *R. leguminosarum* sv. *viciae* strains nodulating *Vicia cracca* plants correlated with differences in soil pH and geographical locations. Also, Zhang et al.^60^ confirmed the influence of soil types and altitude on the biogeographical patterns of rhizobial strains originating from seven sites in Northwest China. Moreover, genetic characterization of red clover isolates from two Carpathians regions in Romania (in total 60 strains) indicated that differences in the chromosome composition were related to the geographical distance and depended on altitude, whereas the diversity in the composition of plasmid sequences was affected by both soil pH and altitude^39^. Palmer and Young^61^ found a higher genetic diversity of *R. leguminosarum* sv. *viciae* populations in arable soils than in grass soils, indicating that long-term cultivation of pea (*Pisum sativum*) can positively change bacterial diversity in soil. This suggests that rhizobial diversity can be affected by differences between these two management regimens. In addition, they found that the lower diversity was associated with high potential nitrogen and phosphate levels in soil or soil acidity.

Interestingly, the occurrence of *R. leguminosarum* sv. *trifolii* strains nodulating three *Trifolium* species (i.e., *T. pratense, T. repens*, and *T. hybridum*) was confirmed in Arctic and subarctic regions of Norway (from 78° to 60°N)^35^. The authors characterized microsymbionts of these plants grown in Piramiden in Svalbard islands (Arctic zone) and in north (Tromso) and south (Bergen, Valdres) Norway (subarctic zone). In total, 243 *R. leguminosarum* sv. *trifolii* isolates from these three clover species were ERIC-PCR fingerprinted and 56 distinct patterns were found, which were associated to the localities where these bacteria were trapped (no similar ERIC-PCR patterns were found in soils from different sites). They indicated that, in the extreme conditions in the Arctic, rhizobia survived as saprophytes and in symbiosis with clovers. The chromosomal diversity of these populations mapped by rep-PCR demonstrated that the separation of chromosomal types was influenced by their geographical origin^35^. Furthermore, the occurrence of strains closely related to *R. leguminosarum* sv. *trifolii* was confirmed even in such extreme conditions as arctic regions. The presence of *R. leguminosarum* sv. *viciae* strains nodulating *Lathyrus japonicus* and *Lathyrus pratensis* plants grown in northern Quebec (Cananda) was reported. However, this study was done using only a low number of rhizobial strains. Interestingly, these bacteria showed different capacities for growing at low temperatures (including isolates that were able to grow even at such a low temperature as 5 °C)^36,37^. These data indicate that rhizobial strains belonging to different symbiovars of the *R. leguminosaum* species are able to exist in various geographical regions with highly stressful conditions, such as the low temperatures in the arctic and subarctic zones. This confirms the high adaptation potential of the strains from this rhizobial species. As evidenced, the adaptation of these bacteria to low temperature stress is ensured, among others, by mechanisms related to the production of cold shock (CSP) and cold adaptation proteins (CAP) as well as the synthesis of unsaturated fatty acids^37^. Since cold-adapted rhizobia isolated from arctic or subpolar regions were able to improve symbiotic nitrogen-fixation and yields of legumes in low temperature conditions^62,63^, they are an interesting objective for both studying and searching valuable strains for future potential agriculture applications.

In this study, we additionally constructed a phylogeny between the representative strains from both populations based on both single and concatenated sequences of five house-keeping genes (*atpD, rpoB, glnII, recA*, and *gyrB*). Our results of MLSA with 30 representative strains (15 strains per each collection) demonstrated a high degree of heterogeneity within the *R. leguminosarum* sv. *trifolii* strains analyzed. These data are in congruence with those published earlier^39,44,59–61^ and recently by Young and colleagues in a paper^57^, in which comprehensive analysis of 429 publicly available genome sequences of *R. leguminosarum* strains was performed. The authors have suggested that bacteria currently included in *R. leguminosarum* are too diverse to be considered a single species; therefore, they referred to this as a species complex (Rlc). They constructed a phylogeny based on concatenated sequences of 120 core genes, which allowed identification of 18 distinct genospecies within Rlc, plus 7 unique strains that were not placed in these genospecies^57^. Among them, genospecies C (including 147 strains), E (79), B (45), A (38), N (12), and R (12) were represented most frequently, whereas the remaining genospecies were less abundant: D, O, and Q (each included 8 strains), M (6), H, I, and K (5 each), L, G, and S (3 each), J and P (2 each). A few years earlier, Young’s group in an excellent paper^30^ proposed idea of genospecies occurring within highly differential *R. leguminosarum* species. Kumar et al. confirmed in this study a high diversity of *R. leguminosarum* isolates obtained from nodules of *V. sativa* and *T. repens* plants, grown on as small area as 1 m^2^ of road-side verge in Yorkshire, UK. They identified 72 isolates, which based on a concatenated sequence of 305 conserved core genes and ANI (Average Nucleotide Identity) parameter were sufficiently diverged to be recognized as separate genospecies (named as gsA-E). Interestingly, different effects of interactions between these strains were detected (growth stimulation or suppression), depending on their genospecies (e.g., gsE showed the highest inhibition capacity, whereas gsA the highest susceptibility)^64^. Moreover, Young and others showed that three house-keeping gene sequences (*atpD*, *gyrB*, and *recA*) are sufficient to assign strains to individual genospecies^57^. Based on these data, using the concatenated sequences of the *atpD, rpoB, glnII, recA*, and *gyrB* genes, we classified the representatives of the two climate populations to four genospecies (A, B, K, and E). The temperate strains were assigned to three genospecies (B, K, and E), whereas the subpolar strains were classified only to two genospecies (A and B). Furthermore, some differences between clover plants, in respect to a number of representative strains isolated from their nodules, as well as rhizobial genospecies, were detected. In the case of plants coming from the subpolar region, comparable frequency of occurrence of strains from gsA and gsB in nodules of individual plants was found. A little higher diversity was observed between plants coming from the temperate region; five representative strains (belonging to gsB and gsK) were isolated from nodules of plant 1, whereas only one representative (gsK) from each plants 3 and 4. Among the temperate strains, genospecies B was dominant, whereas gsA was absent. These data may reflect the influence of local environmental conditions, including low temperature, on the rhizobial diversity in this geographic region. In general, a great majority of the studied strains belonged to the frequently occurring gsB and gsA, which was in line with the data presented by Young et al.^57^. However, we identified only 2 strains (8-3 and 10-3) from gsE, which is highly represented among Rlc strains. Interestingly, 3 strains (KW1-9, KW2-9, and 5-8) were assigned to the occasionally occurring gsK, which currently comprises only 5 of the 429 analyzed strains. In summary, the strains coming from the subpolar climate grouped together and formed distinct clades (i.e., one comprising 9 gsA strains and a distinct group within gsB), clearly separated from those formed by the strains from the temperate climate (i.e., gsK and gsE). Only few strains from the two geographical regions with the different temperature conditions formed a common group comprising strains assigned to gsB. These data are in congruence with our results obtained in the fingerprinting analyses.

As indicated, MLSA is a reliable and effective methodology for studying phylogenetic relationships between bacterial strains^65,66^. Usually, at least four house-keeping genes are used in MLSA for phylogenetic studies of the order *Rhizobiales*, e.g., *atpD, dnaK, glnII, gltA, recA, rpoB*, and *thrC*^65–69^. Our results also confirmed that genes *atpD, rpoB, recA*, and *gyrB* are the most reliable and effective for this type of analysis.

Phylogenetic MLSA of native rhizobia nodulating faba bean (*Vicia faba* L.) in Egypt based on concatenated sequences of these genes revealed that a majority of the strains nodulating this legume host belonged to *R. leguminosarum* sv. *viciae*^70^. Similarly, the *glnII, recA, atpD,* and *dnaK* genes proved to be efficient in determination of the phylogeny and taxonomy of a diverse collection of *Bradyrhizobium* strains^65^. However, in contrast to our results and data reported by Stefan and others^37^ on *R. leguminosarum* sv. *trifolii* and *viciae* strains, Menna et al.^65^ observed that the geographical origin of strains did not affect the patterns of their house-keeping genes, reinforcing the conviction of a common origin for *Bradyrhizobium* with subsequent diffusion of the strains by soil-contaminated seeds.

In conclusion, the concatenated sequence analysis of house-keeping genes is a powerful method to conduct reliable phylogenetic analysis of various rhizobial strains and determine their high intra- and interspecies genetic variations. Furthermore, our results and those earlier published in several papers showed very interesting findings that several distinct *R. leguminosarum* genospecies coexist at one site, and the same genospecies are found in other regions where the local conditions are substantially different. For example, gsA was also found in Australia, Greece, India, and USA, gsB in Greece, Germany, China, and Peru, gsC in Australia, gsD in USA, gsE in Russia, Italy, USA, Peru, and Ethiopia^30,57,70–73^. All these data indicate that strains belonging to *R. leguminosarum* species are widespread.

## Materials and methods

### Sampling of root nodules, bacterial isolation, and nodulation tests

Root nodules of red clover used in this study were sampled from two European regions (Poland, Lublin region, 51°15′55–57″ N, 22°32′6-10″ E and Norway, Tromsø region, 69°38′36-40″ N, 18°54′ 00-01″ E) in June 2016. The sampling sites were located in meadows with no history of rhizobial inoculation. The samples were collected in the same way to minimize the effects of different environmental factors. Five plants per region were sampled and the distance between them was 20 m (sampling pattern was along a straight 80-m long line). For this, the plants were dug up using a hand hoe, placed in plastic bags containing wet cotton wool, and transported to the laboratory. The harvested plants were confirmed as wild red clover (*Trifolium pratense*), for which no official and national permissions for collection were needed. We stated that our study complies with relevant institutional, national, and international guidelines and legislation. The roots were washed, and nodules were detached and stored in sterile plastic vials with wet cotton wool at 4 °C prior to bacterial isolation. 20 nodules were randomly selected from each plant. Standard routine laboratory techniques were applied for isolation of bacteria from the nodules^33,34^. Briefly, the roots were washed with water to remove soil particles, placed in 70% ethanol for surface sterilization, treated with 0.1% HgCl_2_, and rinsed with sterile water. Then, the nodules were crushed, spread onto 79CA agar plates^74^ and incubated at 28 °C for 4 days. As indicated by the appearance and color of the bacterial colonies, a number of isolates were not rhizobial strains, but were probably endophytes or contaminants, and were not further analyzed. Finally, a total of 120 rhizobial strains were isolated in pure culture (60 strains from each climate collection). For further experiments, the isolated strains were maintained in 79CA medium with 1% (w/v) glycerol as a carbon source at 28 °C with shaking (160 rpm). Bacterial isolates used in this study are listed in Table 2.

The nodulation capability of the strains was tested by inoculating seedlings of *T. pratense* (L.) (cultivar Dajana). For this experiment, 20 glass tubes containing single clover seedlings growing in Fahraeus nitrogen-free agar^75^ were used for each strain and cultivated in plant growth chamber (25 °C, 80% humidity) during four weeks. Nodulation capacity was recorded for each strain as positive (Nod +) or negative (Nod-) depending on the presence or absence of nodules on roots. Nitrogen fixation was considered effective when nodules were pink (Fix +) and ineffective if nodules were white (Fix-).

### Genomic analyses of strains isolated from red clover nodules

#### Isolation of total DNA and sequence analysis of the 16S rRNA gene

For isolation of total DNA from the studied strains, 5 ml of 24-h bacterial cultures in 79CA medium and the guanidium thiocyanate extraction method was used^79^. DNA concentration and purity in the samples were assessed using a Nanodrop 2000/2000c (Thermo Scientific, USA). In order to classify the strains to particular species, sequence analysis of the 16S rRNA gene was performed. In this approach, a nearly full-length gene (up to 1309 bp) was PCR amplified and sequenced using primers fD1d and rPla (Table 3) under the following conditions: initial denaturation at 94 °C for 3 min; 35 cycles of 1 min at 94 °C, 1 min at 55 °C, 2 min at 72 °C; followed by a final 7-min elongation step at 72 °C^80^. Each PCR was carried out in a total volume of 100 µl and the mixtures contained 5 µl of template DNA (100 ng/µl), 1 µl of each primer (10 pmol/µl), 50 µl polymerase reaction buffer (ReadyMix Taq kit, Sigma-Aldrich, USA), and 43 µl of milli-Q water. The PCR reactions were performed in a thermocycler (Biometra, T-48 Personal, Germany). Next, PCR amplicons obtained were purified using a Clean-up kit (A&A Biotechnology, Poland) and sequenced using the BigDye terminator Cycle Sequencing kit and the 3500 Genetic Analyzer according to the manufacturer’s protocol (Applied Biosystems, USA). Sequencing was carried out by Genomed Company (Poland). All sequences of the 16S *rRNA* gene were deposited in the GenBank database under accession numbers OL451244–OL451302, OL453214–OL453270, OL546809–OL546813 and OL546815.

**Table 3:** Oligonucleotide primers used in this study.

Name	Sequence (5’-3’)	Target	References
fD1d	GAGAGTTTGATCCTGGCTCAGA	16S rRNA	^80^
rPla	CTACGGCTACCTTGTTACGACTT	16S rRNA	^80^
FGPS1490	TGCGGCTGGATCACCTCCTT	16S-23S rRNA ITS	^81^
FGPL132’	CCGGGTTTCCCCATTCGG	16S-23S rRNA ITS	^82^
ERIC-1	CACTTAGGGGTCCTCGAATGTA	repetitive intergenic genomic regions	^83^
ERIC-2	AAGTAAGTGACTGGGGTGAGCG	repetitive intergenic genomic regions	^83^
BOX1AR	CTCCGGCAAGGCGACGCTGAC	genomic repetitive sequences	^84^
recA640R	ACATSACRCCGATCTTCATGC	*recA*	^85^
recA41F	TTCGGCAAGGGMTCGRTSATG	*recA*	^85^
glnII689	TGCATGCCSGAGCCGTTCCA	*glnII*	^85^
glnII12F	YAAGCTCGAGTACATYTGGCT	*glnII*	^85^
atpD871R	AGMGCCGACACTTCMGARCC	*atpD*	^86^
atpD352F	GGCCGCATCATSAACGTSATC	*atpD*	^86^
gyrB1043R	AGCTTGTCCTTSGTCTGCG	*gyrB*	^87^
gyrB343F	TTCGACCAGAAYTCCTAYAAGG	*gyrB*	^87^
rpoB1346	TCGATGTCGTCGATYTCGCC	*rpoB*	^88^
rpoB454F	ATCGTCTCGCAGATGCACCG	*rpoB*	^88^

#### PCR-based restriction fragment length polymorphism of the 16S–23S rRNA intergenic transcribed spacer

16S-23S rDNA ITS was amplified using primers FGPS1490 and FGPL132’ (Table 3), which corresponded to 1521–1541 bp and 114–132 bp positions of this genomic region in *E. coli*, respectively. Amplification reactions were carried out in a final volume of 50 µl, which contained 2.5 µl of template DNA (100 ng/µl), 5 µl of each primer (10 µM/µl), 25 µl of ReadyMix Taq kit (Sigma-Aldrich, USA), and 12.5 µl of milli-Q water. PCR was performed using the following protocol: initial denaturation at 95 °C for 3 min, followed by 35 cycles of 1 min at 94 °C, 1 min at 50 °C, 2 min at 72 °C, and a final 3-min elongation step at 72 °C. Depending on the strain, amplicons of the length from ~ 1200 to 1300 bp were obtained. These PCR products were purified with Clean-up kit (A&A Biotechnology, Poland) and digested with one of the 4-bp restriction endonucleases *Msp*I*, Taq*I*,* and *Bsu*RI (Thermo Fisher Scientific, USA) according to the manufacturer’s recommendations. In this step, 0.5 µg of DNA in a total reaction volume of 20 µl was applied. The restriction fragments were separated in 3% (w/v) agarose gels containing Simply Safe (EURx, Poland) for DNA detection. Electrophoresis was carried out using 1xTBE buffer at 100 V for 6 h. The restriction patterns were visualized using UV light and documented (Quantum-Capt, Vilber, France). A 1-kb ladder (GeneRuler DNA Ladder Mix, Thermo Fisher Scientific, USA) was used as a molecular size marker. The genetic relationships between the studied strains were determined by calculation of the Nei and Li (Dice) coefficient^89–91^ and a dendrogram was prepared according to the unweighted pair group method with arithmetic averages (UPGMA)^92^ using BIO1D v. 11.10 program.

#### Analysis of BOX-PCR and ERIC-PCR patterns

Repetitive element sequence-based PCR analyses (rep-PCR) were performed using Enterobacterial repetitive intergenic consensus primers ERIC-1 and ERIC-2 (for ERIC-PCR) and the Enterobacterial repetitive sequence BOX1AR primer (for BOX-PCR)^78,79^. The ERIC-PCR mixtures contained 100 ng of DNA, 1 µl of ERIC-1, 1 µl of ERIC-2 (10 pmol/µl), 10 µl of ReadyMix Taq (Sigma-Aldrich, USA), and 7 µl of milli-Q water. The BOX-PCR mixtures contained 100 ng of DNA, 2 µl of primer BOX1AR (10 pmol/µl), 10 µl of ReadyMix Taq and 7 µl of milli-Q water. The amplification cycle was as follows: initial denaturation at 94 °C for 5 min, followed by 35 cycles of denaturation at 94 °C for 1 min, annealing at 53 °C (for BOX-PCR) or 49 °C (for ERIC-PCR) for 1 min, elongation at 65 °C for 6 min, and final elongation for 10 min at 65 °C. The PCR products were separated by electrophoresis in 1.5% (w/v) agarose gels for 1.5 h at 100 V and visualized under UV light; next, the profiles were collected for further analysis. The number and size of the amplicons obtained for individual strains were determined and band patterns were grouped using the Nei and Li (Dice) coefficient^89,90^. Next, the dendrogram was constructed using the BIO1D v. 11.10 program with the unweighted pair group method with arithmetic averages (UPGMA). Based on the results obtained from both the ERIC-PCR and BOX-PCR analyses, isolates representing different clusters were chosen for the multi-locus sequence analysis of house-keeping genes.

#### Multi-locus sequence analysis (MLSA) of house-keeping genes

All PCR reactions were performed in a total 100-µl volume, which contained 200 ng of genomic DNA, 1 µl of each primer (10 pmol/µl), 50 µl of ReadyMix Taq kit and 43 µl of milli-Q water. A recA640R and recA41F primer set (Table 3) was used to amplify a 495-bp internal fragment of *recA* (for recombinase A)^85^. The PCR cycle was as follows: initial denaturation at 95 °C for 5 min; 30 cycles of 1 min at 94 °C, 40 s at 60 °C, 90 s at 72 °C, followed by a final 5-min elongation step at 72 °C. A partial sequence of *glnII* (for glutamine synthase II) with the length of 618 bp was amplified using primers glnII689 and glnII12F^85^ and the same PCR protocol as for the *recA* gene. An internal fragment of the *atpD* gene (for the ATP synthase β subunit) with the length of 432 bp was amplified using primers atpD871R and atpD352F^86^. A gyrB1043R and gyrB343F primer set was used for amplification of the *gyrB* fragment (for the gyrase B protein subunit) of the length 654 bp^87^. The PCR cycle conditions for the amplification of *atpD* and *gyrB* were identical as for *recA*, except for the annealing temperature (55 °C for *atpD* and 45 °C for *gyrB*). Partial sequences of *rpoB* with the length of 855 bp (for the RNA polymerase β subunit) were amplified with primers rpoB1346 and rpoB454F^88^, using the same conditions and reaction composition as for *recA,* except for the annealing temperature (63 °C). The PCR products were analyzed by electrophoresis in 1% agarose gels, purified using Clean-up kit (A&A Biotechnology, Poland), and then sequenced using the BigDye terminator Cycle Sequencing kit and the 3500 Genetic Analyzer according to the manufacturer’s protocol (Applied Biosystems, USA). Sequencing was carried out by the Genomed Company (Poland). The sequences of the house-keeping genes were deposited in the GenBank database under accession numbers OL555858-OL555887 for *recA*, OL555888–OL555917 for *atpD,* OL555828–OL555857 for *gyrB*, OL555798–OL555827 for *glnII*, and OL555918–OL555947 for *rpoB*. The entire list of accession numbers of these genes can be found in Supplementary Table S4 online.

#### Construction of phylogenetic trees

For phylogenetic analyses, the nucleotide sequences obtained in this study were compared with those obtained from the National Center for Biotechnology Information (NCBI) database using the BLASTN program^92^. Then, the sequences of the studied strains and the sequences available in the databases were aligned using the ClustalX software^93^ and corrected manually using GeneDoc^94^. The phylogenetic trees of the individual chromosomal genes (*atpD, recA, gyrB, rpoB,* and *glnII*) and the MLSA tree based on the concatenated sequences of these genes were constructed with the Maximum-Likelihood (ML) method using the best DNA substitution model determined in MEGAX. For MLSA, a combined 3,054-bp sequence, which was composed of 432 bp of *atpD*, 495 bp of *recA*, 855 bp of *rpoB*, 654 bp of *gyrB*, and 618 bp of *glnII,* was used. The phylogenetic distances between the studied strains generated from the concatenated sequences of the genes (*atpD* + *recA* + *rpoB* + *gyrB* + *glnII*) were determined using the General Time Reversible (GTR) model with invariable-sites-plus-gamma (+ I + G)^95^. In contrast, the phylogenetic distances for the *recA* and *atpD* genes were calculated according to the Tamura-Nei + I + G model^96^. The reliability of tree topologies was estimated by a bootstrap confidence analysis based on 1000 resamplings^97^. The phylogenetic trees were constructed using the MEGAX software package^98^.

#### Nucleotide sequence accession numbers

All sequences obtained in this study were deposited in the GenBank database and are now publicly available. Sequences of the 16S *rRNA* gene for 120 strains are now publicly available under accession numbers OL451244–OL451302 and OL546815 (strains from the temperate climate), OL453214–OL453270 and OL546809–OL546813 (strains from the subpolar climate), respectively. The sequences of five house-keeping genes for 30 representatives of the two populations were deposited in the GenBank database under accession numbers OL555858-OL555887 (*recA*), OL555888–OL555917 (*atpD*)*,* OL555828–OL555857 (*gyrB*), OL555798–OL555827 (*glnII*), and OL555918–OL555947 (*rpoB*), and are currently publicly available. Accession numbers of house-keeping genes for individual 30 representatives are listed in Supplementary Table S4.

### Ethics approval

This article does not contain any studies with human participants and/or animals performed by any of the authors. The formal consent is not required in this study.

### Statement for plant material

Our study complies with relevant institutional, national, and international guidelines and legislation.

## Supplementary Information

Supplementary Information.

## Data Availability

All sequence data that support the findings of this study have been deposited in GenBank (https://www.ncbi.nlm.nih.gov/genbank/) with accession numbers OL451244.1-OL546813.1 and OL555798–OL555947 and are now publicly available.
