# Supplementary material for: Genetic diversity of microsymbionts nodulating Trifolium pratense in subpolar and temperate climate regions
Source: Sci Rep. 2022 Jul 15;12:12144. doi: 10.1038/s41598-022-16410-0 (PMC9287440; doi:10.1038/s41598-022-16410-0)

SUPPLEMENTARY MATERIAL

Genetic diversity of microsymbionts nodulating *Trifolium pratense* in subpolar and temperate climate regions

Marta Kozieł [1], Michał Kalita [2] and Monika Janczarek [1,*]

[1] Department of Industrial and Environmental Microbiology, Institute of Biological Sciences, Faculty of Biology and Biotechnology, Maria Curie-Skłodowska University, 19 Akademicka, 20-033 Lublin, Poland;

[2] Department of Genetics and Microbiology, Institute of Biological Sciences, Faculty of Biology and Biotechnology, Maria Curie-Skłodowska University, 19 Akademicka, 20-033 Lublin, Poland;

***** *Correspondence to:*

Monika Janczarek, E-mail: monika.janczarek@mail.umcs.pl

ORCID identifier: 0000-0002-2250-6358

Table S1. ERIC-PCR patterns and size of fragments obtained among *R. leguminosarum* sv. *trifolii* strains

Pattern number	Strains	Size of fragments (bp)
1	R1	317 447 482 579 1,259
2	R3	234 412 579 740 794 1,259
3	R5 R139	205 294 402 482 546 611 794 829 1,162 1,259 1,771 2,032 2,420
4	R6 R9 R11 R12 R25 R30 R31 R34 R58 R59 R70 R112 R118 R119 R120 R127	205 294 391 482 546 579 611 794 1,207 1,259
5	R10	205 243 335 391 412 579 611 648 740 794 1,259
6	R13	171 261 280 412 482 520 546 740 1,062 1,162 2,032
**7**	R16 R53 R55 R101 R111 R114 R116 R122 R133 R141	205 294 391 482 546 579 611 794 1,162 1,259 1,771
8	R17	205 243 391 412 579 611 648 740 794 1,259
9	R23	205 280 335 482 520 579 648 794 1,207 1,259 1,671 2,134 2,500
10	R26	205 234 280 447 579 611 719 794 872 1,207 1,259
11	R32 R65 R107	579 611 794
12	R33	234 335 546 579 740 1,162
13	R37	294 335 412 611 648 740 794 1,259
14	R39	294 412 611 740 794 1,207 1,259 1,771
15	R41	310 335 520 579 681 1,259 1,771
16	R43	205 294 391 482 520 579 611 794 1,162 1,259 1,771 2,500
17	R49	310 520 579 611 740 1,062 1,162
18	R51	294 310 482 794
19	R56	280 355 447 482 532 546 611 719 1,259 1,771
20	R66	171 280 520 719
21	R68	391 482 546 611 740 1,062 1,259 2,500
22	R108	234 280 447 520 611 740
23	R115	205 280 355 471 532 546 611 719 794 1,259 1,771
24	R121	205 243 391 447 482 579 611 740 794 872 1,259
25	R124	205 280 447 579 611 719 794 872 1,207 1,259
26	R130	205 294 412 482 520 546 579 611 719 794 1,207 1,259
27	R137	171 280 391 482 546 611 719 829 955 1,207 1,259 1,771 2,032
28	R140	205 280 335 482 508 579 648 794 1,207 1,259 1,671 2,134 2,500
29	2-1	261 494 546 579 611 719
30	2-2	280 508 589 719
31	2-4 9-5	171 280 520 740
32	3-1	171 294 520 740 1,102 1,162 1,207
33	3-2	294 520 740 1,102 1,162
34	3-3	520 611 719 1,062
35	3-4 4-3	171 391 447 482 546 579 719 740 794 1,062
36	4-1	171 234 261 482 681
37	4-2	171 391 447 482 546 579 719 740 794 1,102
38	4-4	171 391 494 719 1,102
39	5-1	520 611 719 1,062 2,032
40	5-4	171 280 520 546 579 740 1,207 2,032 2,420
41	5-5	310 391 412 447 494 520 648 682 1,259 1,535 2,905
42	5-8	234 310 370 391 412 520 546 719 794 812 1,062 1,771 2,500
43	6-1	181 234 280 310 412 447 520 546 579 740 794 872 1,062 2,032 2,420
44	6-4	391 740 1,162 2,032

45	6-6	181 234 294 494 719
46	6-7	181 294 494
47	6-9	171 280 391 508 719 1,062 1,207
48	6-11	280 520 740 1,162
49	6-12	294 740 1,259
50	6-15	412 520 740 1,062 1,259
51	7-7	171 261 391 719
52	8-2	280 380 391 447 508
53	8-3	355 391 447 648 1,434
54	8-8	520 611 719 1,062 1,162
55	8-11	520 1,162 1,535 2,032
56	8-12	171 191 234 261 310 355 412 471 520 546 681 1,207 1,535
57	9-4	261 310 447 471 546 719 1,612
58	10-3	370 520 648 794 1,207 1,771 1,883 2,032
59	10-4	280 508 579
60	10-12	294 412 520 719 740 812
61	KW1-4	294 447 520 740 1,102 1,162
62	KW1-5	181 294 310 391 447 520 740 1,162 2,500
63	KW1-8	171 181 355 391 508 719
64	KW1-9	647
65	KW1-10	171 181 294 412 508 611 681 719 812 1,102
66	KW2-2	171 234 280 412 520 719 740 812
67	KW2-3 KW2-10	234 280 508 611
68	KW2-4	181 294 520 740 1,102 1,162
69	KW2-6	579 681
70	KW2-7	181 294 520 740 1,102 1,162 1,207
71	KW2-9	611
72	KW2-12	261 355 447 494 520 719 1,102
73	M1	171 234 280 294 494 520 546 579 740 794 872 1,162 1,207 1,570
74	M2	494 740
75	M4	171 391 740 1,102
76	M6	261 370 494 740 872
77	M8	520 740
78	M10	171 234 261 494 520 546 579 740 794 872 1,162 1,20 1,570
79	M13	171 261 494 740 1,102
80	M14	171 261 494 740
81	M15	171 261 494 546 740 1,102
82	M16	171 205 261 494 579 740 1,102
83	M17	171 391 719 1,102
84	M19	171 261 494 719
85	M20	171 391 494 740 1,102 1,162 1,771
86	24.2	171 191 355 391 447 520 648 995 1,535 1,771 2,500
87	TA1	181 234 280 508
88	3841	412 520 794 812 955 1,102 1,259
89	VF39	719 1,771 2,500

Table S2. BOX-PCR patterns and size of fragments obtained among *R. leguminosarum* sv. *trifolii* strains

Pattern number	Strains	Size of fragments (bp)
1	R1	335 411 480 519 719 743 815 855 3,633
2	R3	355 411 480 583 719 743 815 1,050 1,345
3	R5	335 583 743 815
4	R6 R11 R12	335 411 542 583 719 743 815 1,050 1,580 2,230 2,500
5	R9	335 411 542 583 719 743 815 1,050 1,580 1,823 2,230 2,500
6	R10 R17	335 411 583 641 669 719 815 1,050 1,345 1,580
7	R13	335 411 815 1,050 1,208
8	R16 R101	280 335 411 719 743 815 2,230 2,500
9	R23	335 411 542 583 719 743 815 855 1,580 3,633
10	R25	335 743 815 1,050
11	R26	335 411 445 560 719 743 815 1,345
12	R30 R31 R58 R59	280 310 335 411 542 560 719 743 815 1,050 1,580 2,230 2,500
13	R32	260 743 811
14	R33 R49	335 411 793 815 1,050 1,208
15	R34	335 411 445 542 560 719 743 815 3,307
16	R37	335 560 611 641 669 951 1,106
17	R39	280 335 411 542 560 719 743 815 1,050
18	R41	310 335 411 445 542 560 641 743 815 889 951 1,050 1,403 1,580 2,500 3,307
19	R43 R55	280 335 411 542 560 719 743 815 1,050 2,230 2,500
20	R51	280 310 335 411 542 743 815 1,403 1,580
21	R53 R65	335 743 815
22	R56	335 411 519 669 815 951
23	R66	310 411 743
24	R68	280 310 335 411 445 560 719 743 815 1,050 1,208
25	R70	335 411 719 743 815
26	R101	280 335 411 719 743 815 2,230 2,500
27	R107 R111	260 310 335 411 611 719 743 815 2,500
28	R108	335 411 560 611 641 669 815 1,050 1,345
29	R112 R118	280 310 335 411 542 560 719 743 815 1,580 2,230 2,500
30	R114	260 335 411 719 743 815
31	R115	310 335 411 519 669 743 815 951
32	R116	260 310 335 411 719 743 815
33	R119	335 411 719 743 815 1,050
34	R120	280 310 335 411 560 719 743 815 889 1,050 2,500 3,307
35	R121	335 560 611 641 775 1,345 1,500 1,580
36	R122	310 335 411 611 743 815
37	R124	280 310 335 411 519 743 815 1,345 2,230 2,500
38	R127	280 310 335 411 560 743 815 889 1,050 2,500 3,307
39	R130	280 310 335 411 743 815 1,050 1,208
40	R133	280 310 335 411 560 583 743 815
41	R137	280 310 335 411 542 641 743 815 1,345 1,500 2,230 2,500 3,000 3,307 3,633 4,526
42	R139	280 310 335 445 480 542 641 743 815 889 1,050 1,208 1,345 2,230 2,390 3,307 3,633
43	R140	310 411 480 542 611 669 743 815 855 1,345 1,500 2,230
44	R141	310 411 542 583 641 743 815 889 1,050 1,208 1,282 1,500 1,823 2,230 2,500 3,307
45	2-1 10-12	335 411 611 719 743 793 815 1,050 1,208 1,823 3,633
46	2-2	335 411 611 719 743 793 815 1,050 1,208 1,823
47	2-4	411 719 743 793 815 1,050 1,208 1,823
48	3-1 3-2	335 411 480 611 719 743 793 815 889 1,050 1,208 1,823 3,633
49	3-3	335 411 611 743 793 815 1,050 1,208
50	3-4	335 411 583 719 743 793 815 1,050 1,208

51	4-1	583 719 793 815
52	4-2	335 445 583 719 743 793 815 889 1,050 1,823
53	4-3 4-4	335 411 583 669 743 793 815 1,050 1,208
54	5-1	335 411 583 719 743 793 815 1,050 1,823
55	5-4	335 411 611 669 743 793 815 1,050 1,208 1,823 3,633
56	5-5	335 411 519 583 641 793 815 1,823 2,230 3,000 3,633
57	5-8	335 381 411 458 480 583 611 669 743 815 1,050 1,208
58	6-1	335 411 480 519 611 719 743 793 815 889 1,050 1,208 1,823 3,633
59	6-4	335 411 480 611 669 743 793 815 1,050 1,208 1,823
60	6-6	411 611 669 815 1,050
61	6-7	719 793 815 1,050
62	6-9	335 411 611 743 793 815 1,050 1,823
63	6-11	335 583 611 743 793 815 1,050 1,208 1,823
64	6-12	335 411 611 743 793 815 1,050
65	6-15	335 411 480 611 743 793 815 1,050 1,208 1,823
66	7-7	260 335 411 611 743 793 815 1,050 1,208 1,823
67	8-2	411 480 611 815 1,050
68	8-3	335 411 519 611 793 815
69	8-8 8-11	335 411 480 583 743 793 815 1,050 1,208 1,823
70	8-12	335 411 519 583 641 793 815 1,823 2,230 3,000
71	9-4	411 583 611 815 1,050 1,500
72	9-5	335 411 583 611 641 815 1,050
73	10-3	335 411 519 583 641 719 793 815 1,050 2,104
74	10-4	335 411 611 669 743 793 815 1,050 1,208 1,823
75	KW1-4	335 411 583 669 793 1,050
76	KW1-5	335 411 480 583 611 669 743 793 815 1,050 1,823
77	KW1-8 KW2-6	335 560 583 719 793 815 1,050
78	KW1-9	583 815 1,050 1,208
79	KW1-10	335 411 583 743 793 815 1,050 1,208
80	KW2-2	335 411 743 793 815 1,050 1,208
81	KW2-3	335 411 583 793 815 1,050 1,208
82	KW2-4	335 411 583 641 719 793 815 1,050 1,208 1,823
83	KW2-7	335 411 583 669 743 793 815 1,050 1,208 1,823
84	KW2-9 KW2-10	793 815
85	KW2-12	335 719 793 815
86	M1	335 411 519 560 641 743 793 815 1,050 1,106 1,208 1,695
87	M2	560 743 793
88	M4	335 560 743 793 815 1,050 1,208
89	M6	335 411 519 560 611 793 815 1,078
90	M8 M16	335 411 560 793 815 1,050 1,208
91	M10	335 411 519 560 611 793 815
92	M13	335 411 560 743 793 815 1,050 1,208 1,775
93	M14	335 411 519 560 793 815 1,050 1,208 1,775
94	M15	335 411 519 560 793 815 1,050 1,106 1,695
95	M17	335 411 560 641 743 793 815 1,050 1,208
96	M19 M20	335 411 560 743 793 815 1,050 1,208
97	24.2	335 411 519 560 611 793 815 2,000 3,307
98	TA1	335 411 519 560 611 815
99	3841	560 583 815 1,050 1,208 1,345 1,580 2,230
100	VF39	793 815 1,050 1,208

Table S3. RFLP patterns of amplified 16S-23S rDNA ITS and size of fragments obtained for *R. leguminosarum* sv. *trifolii* strains using restriction enzymes *BsuR*I, *Msp*I, and *Taq*I

Enzyme	RFLP pattern of amplified 16S-23S rDNA ITS	Size of restriction fragments (bp)
BsuRI	A	50 65 210 230 271 370
	B	50 65 230 257 271 370
	C	65 248 271 295 370
	D	50 65 210 230 295 370
	E	50 65 210 295 390
	F	50 304 380 475
	G	304 370 475
	H	191 237 264 271 295
	I	50 191 237 295 370
	J	50 264 295 304 370
MspI	A	40 50 150 180 222 280 370
	B	40 50 150 215 222 280 370
	C	40 50 150 215 280 287 370
	D	40 50 151 180 257 280 350
	E	40 50 70 180 235 257 380
	F	40 50 140 150 190 215 265
	G	40 50 150 215 257 280 350
	H	140 222 228 280 294
	I	140 280 360 435
	J	40 50 140 150 215 222 310
	K	40 50 140 150 200 215 280
	L	150 200 248 280
	M	150 215 222 280 320 388
TaqI	A	75 90 150 165 250 273
	B	75 90 150 165 250 320
	C	75 90 150 250 329
	D	75 90 150 250 265 365
	E	75 105 150 205 265 390
	F	75 90 250 285 400
	G	90 285 340 365 400
	H	75 90 340 390 410
	I	75 90 150 250 305 380
	J	75 90 305 340 410
	K	75 90 150 250 350

Table S4. GenBank accession numbers of the genes sequenced in this study and used in MLSA.

Strain	*glnII*	*gyrB*	*recA*	*atpD*	*rpoB*
R1	OL555798	OL555828	OL555858	OL555888	OL555918
R13	OL555799	OL555829	OL555859	OL555889	OL555919
R23	OL555800	OL555830	OL555860	OL555890	OL555920
R26	OL555801	OL555831	OL555861	OL555891	OL555921
R32	OL555802	OL555832	OL555862	OL555892	OL555922
R41	OL555803	OL555833	OL555863	OL555893	OL555923
R49	OL555804	OL555834	OL555864	OL555894	OL555924
R51	OL555805	OL555835	OL555865	OL555895	OL555925
R53	OL555806	OL555836	OL555866	OL555896	OL555926
R56	OL555807	OL555837	OL555867	OL555897	OL555927
R66	OL555808	OL555838	OL555868	OL555898	OL555928
R70	OL555809	OL555839	OL555869	OL555899	OL555929
R108	OL555810	OL555840	OL555870	OL555900	OL555930
R118	OL555811	OL555841	OL555871	OL555901	OL555931
R137	OL555812	OL555842	OL555872	OL555902	OL555932
2-2	OL555813	OL555843	OL555873	OL555903	OL555933
3-1	OL555814	OL555844	OL555874	OL555904	OL555934
3-3	OL555815	OL555845	OL555875	OL555905	OL555935
4-3	OL555816	OL555846	OL555876	OL555906	OL555936
5-8	OL555817	OL555847	OL555877	OL555907	OL555937
6-11	OL555818	OL555848	OL555878	OL555908	OL555938
8-3	OL555819	OL555849	OL555879	OL555909	OL555939
8-11	OL555820	OL555850	OL555880	OL555910	OL555940
10-3	OL555821	OL555851	OL555881	OL555911	OL555941
KW1-9	OL555822	OL555852	OL555882	OL555912	OL555942
KW2-9	OL555823	OL555853	OL555883	OL555913	OL555943
M2	OL555824	OL555854	OL555884	OL555914	OL555944
M14	OL555825	OL555855	OL555885	OL555915	OL555945
M16	OL555826	OL555856	OL555886	OL555916	OL555946
M19	OL555827	OL555857	OL555887	OL555917	OL555947

Figure S1: Dendrogram constructed on the basis of the RFLP analysis of the 16S-23S rDNA ITS using enzyme *Bsu*RI

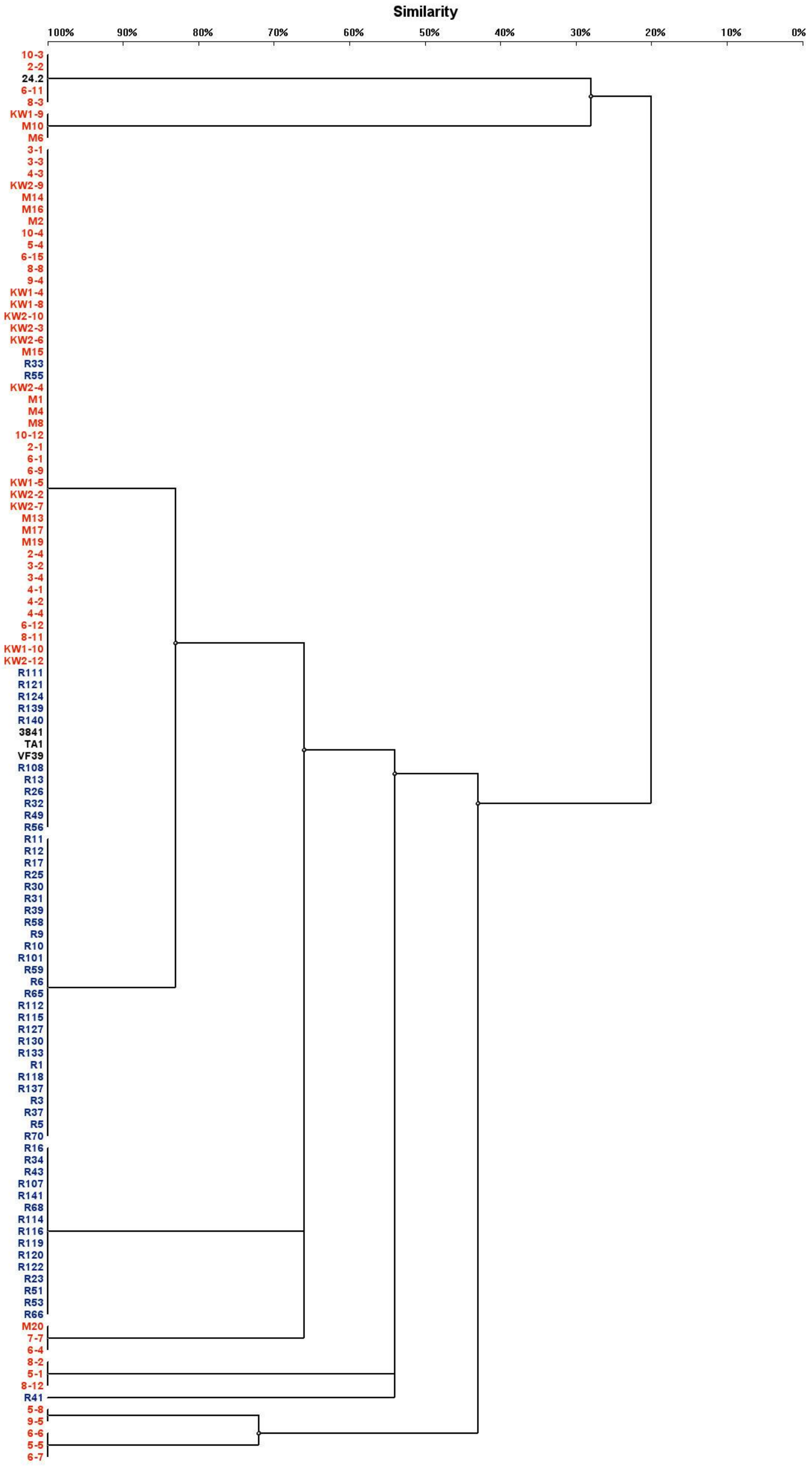

Figure S2: Dendrogram constructed on the basis of the RFLP analysis of the 16S-23S rDNA ITS using enzyme *Msp*I

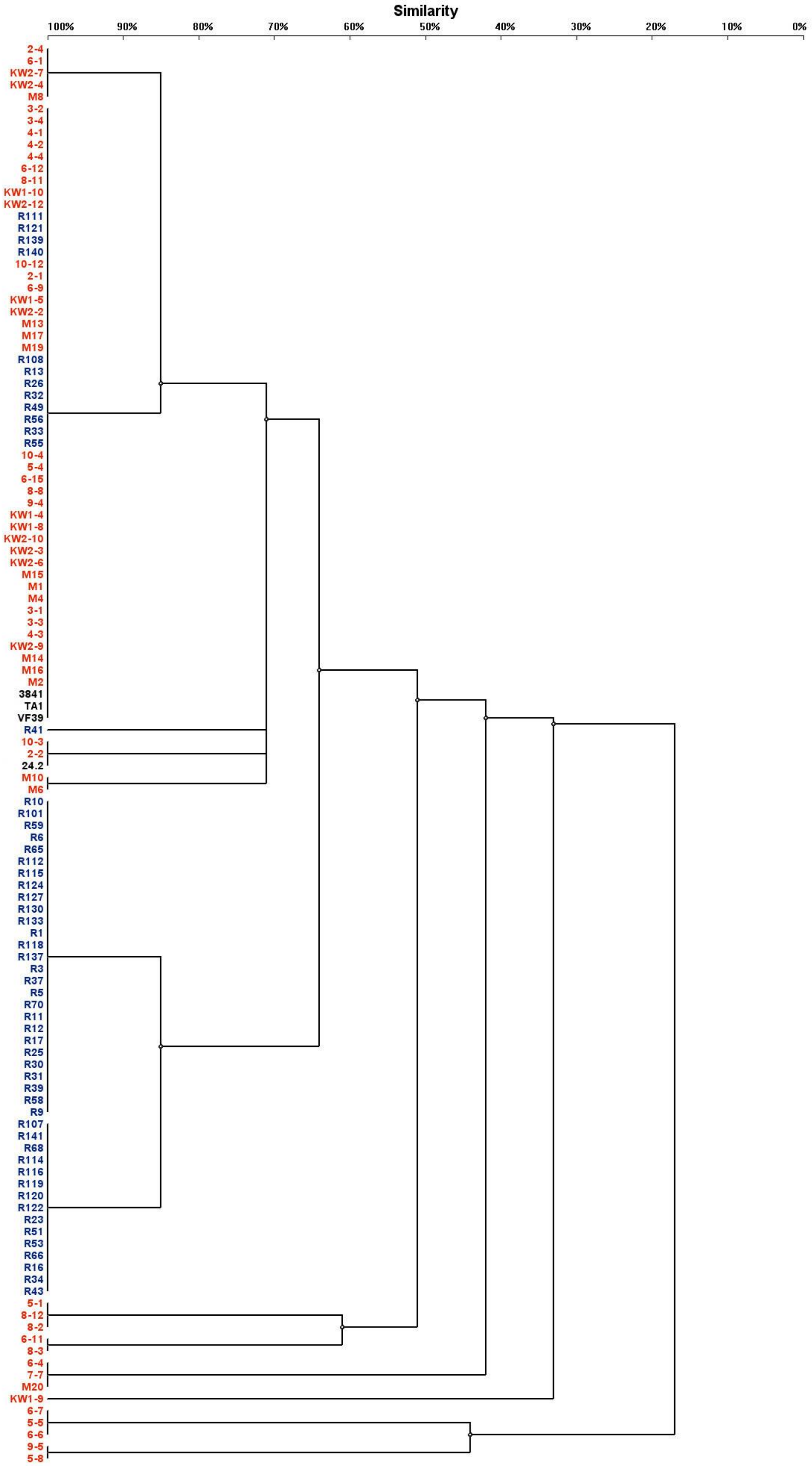

Figure S3: Dendrogram constructed on the basis of the RFLP analysis of the 16S-23S rDNA ITS using enzyme *Taq*I

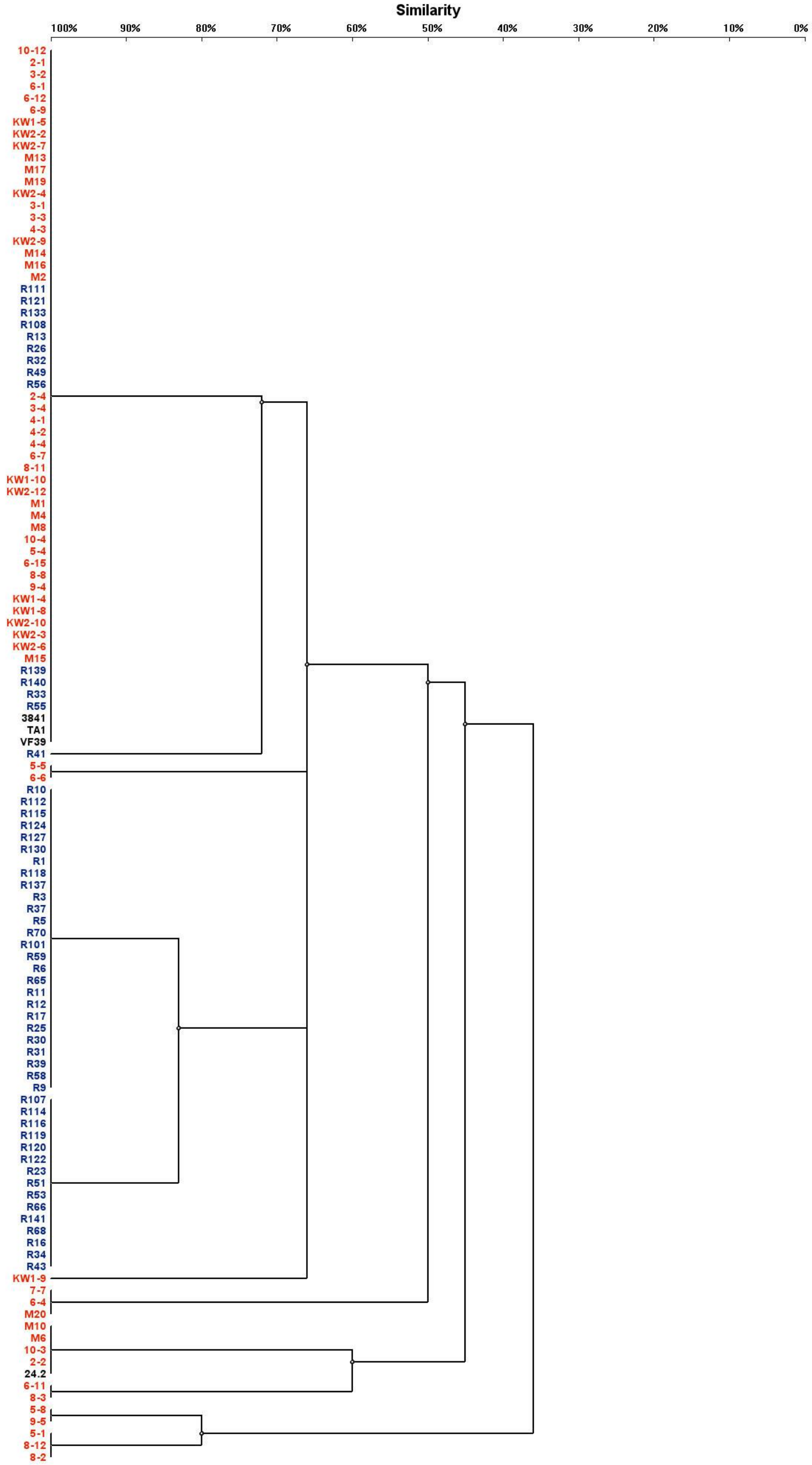

Figure S4: Phylogenetic tree based on the *atpD* sequence (432 bp) showing relationships of the representative red clover isolates with selected reference strains for different rhizobial species. Bootstrap values are shown on the branches. The scale bars represent nucleotide substitutions. Colors indicate genospecies.

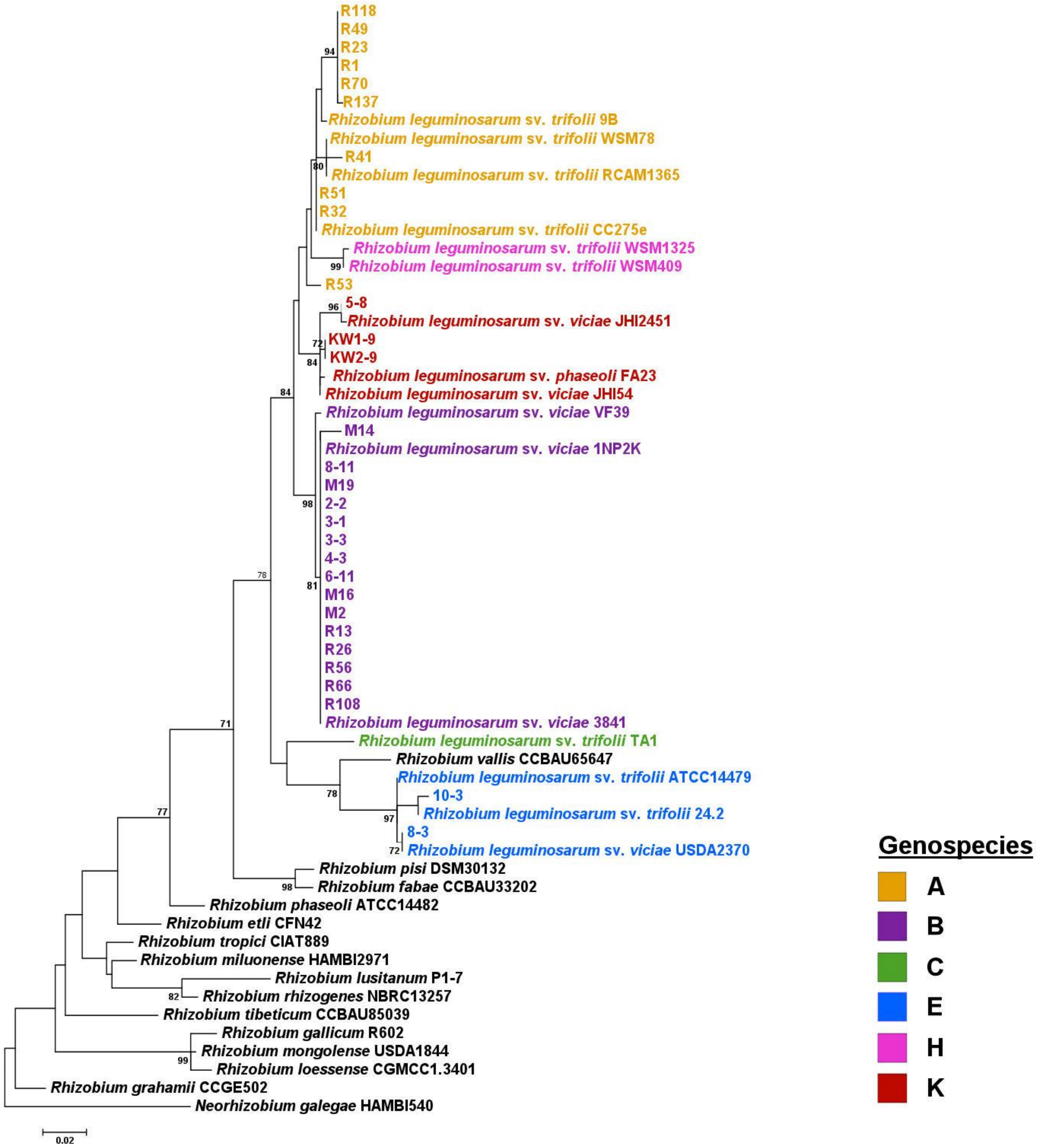

Figure S5: Phylogenetic tree based on the *recA* sequence (495 bp) showing relationships of the representative red clover isolates with selected reference strains for different rhizobial species. Bootstrap values are shown on the branches. The scale bars represent nucleotide substitutions. Colors indicate genospecies.

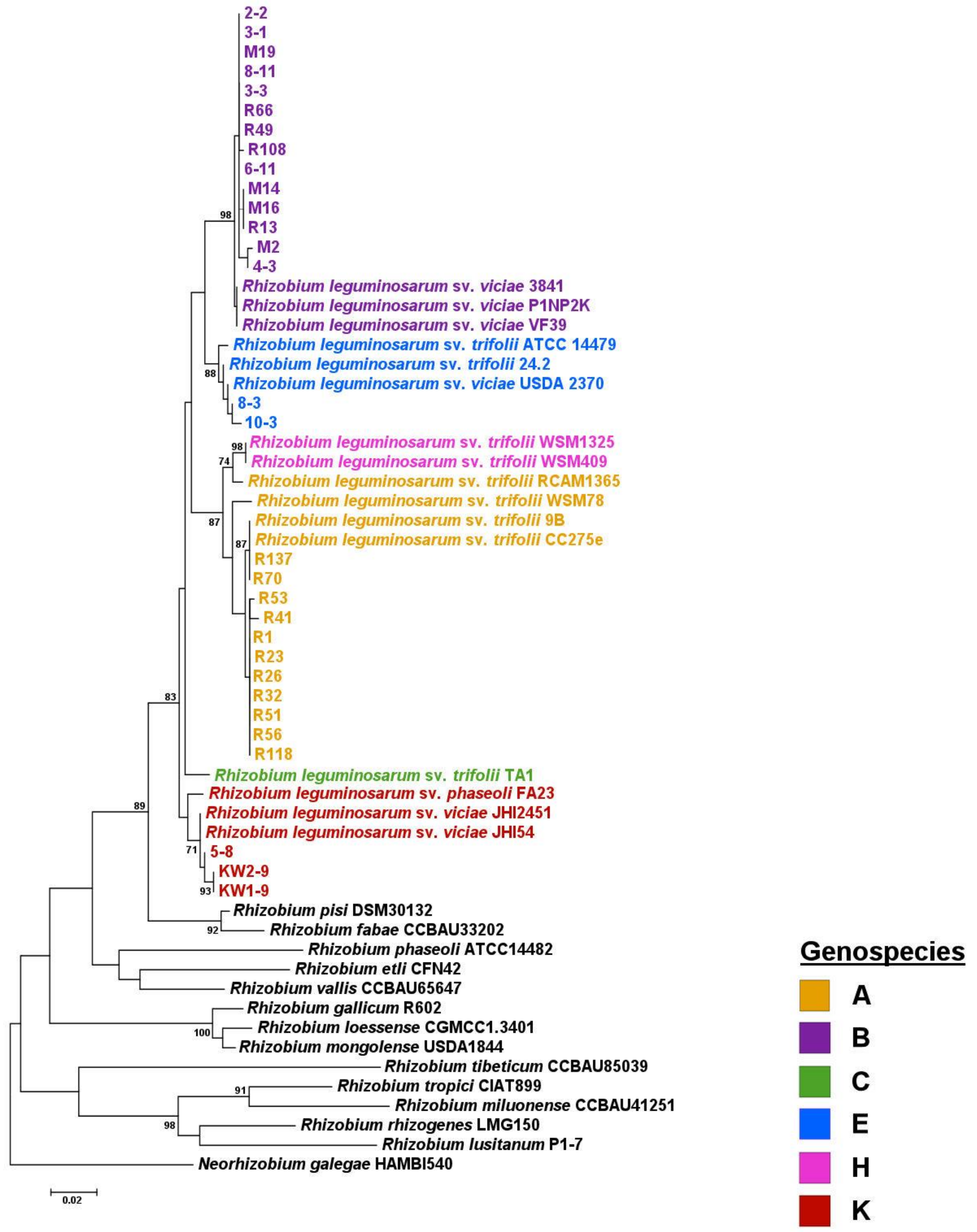

Figure S6: Phylogenetic tree based on the *gyrB* sequence (654 bp) showing relationships of the representative red clover isolates with selected reference strains for different rhizobial species. Bootstrap values are shown on the branches. The scale bars represent nucleotide substitutions. Colors indicate genospecies.

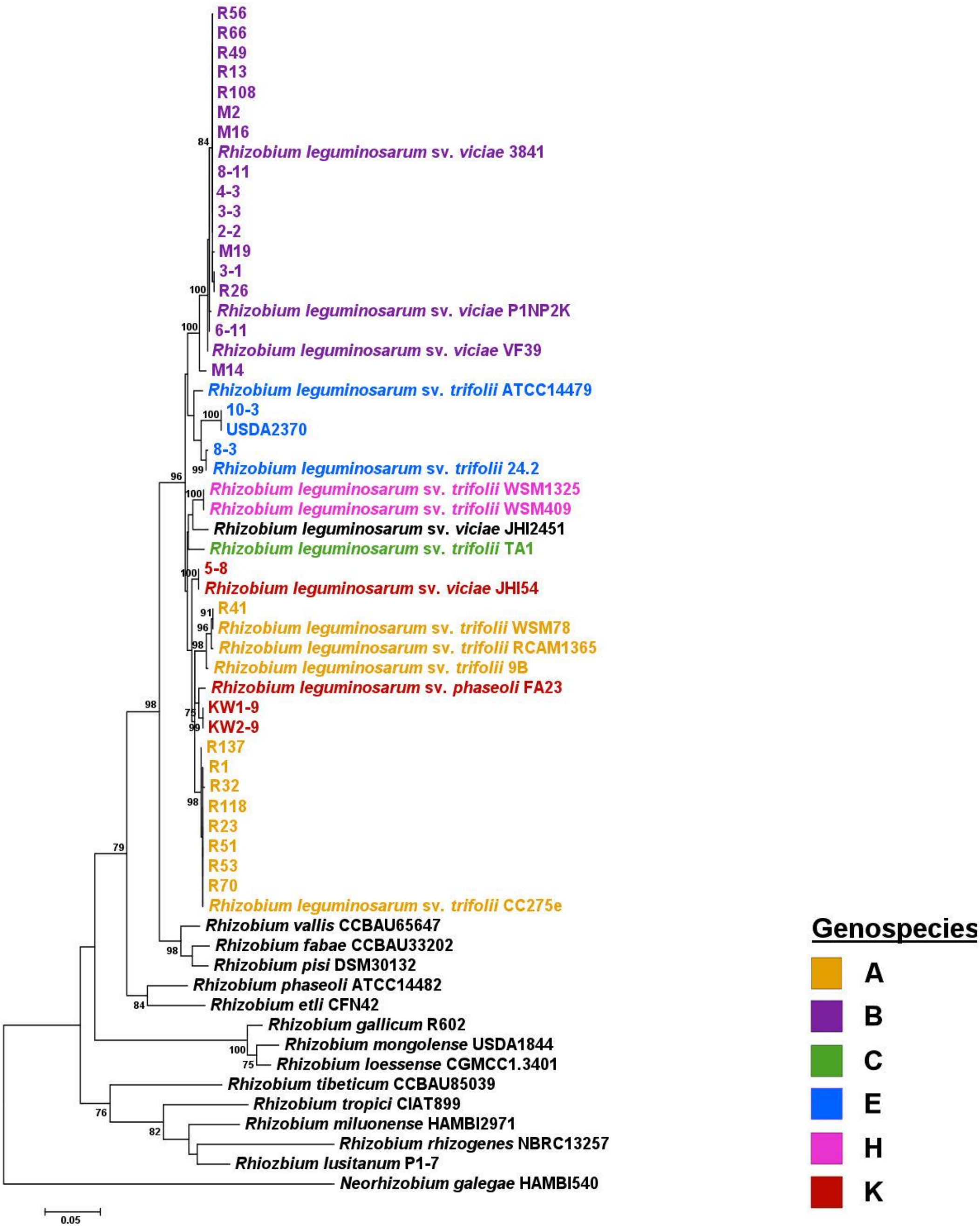

Figure S7: Phylogenetic tree based on the *rpoB* sequence (855 bp) showing relationships of the representative red clover isolates with selected reference strains for different rhizobial species. Bootstrap values are shown on the branches. The scale bars represent nucleotide substitutions. Colors indicate genospecies.

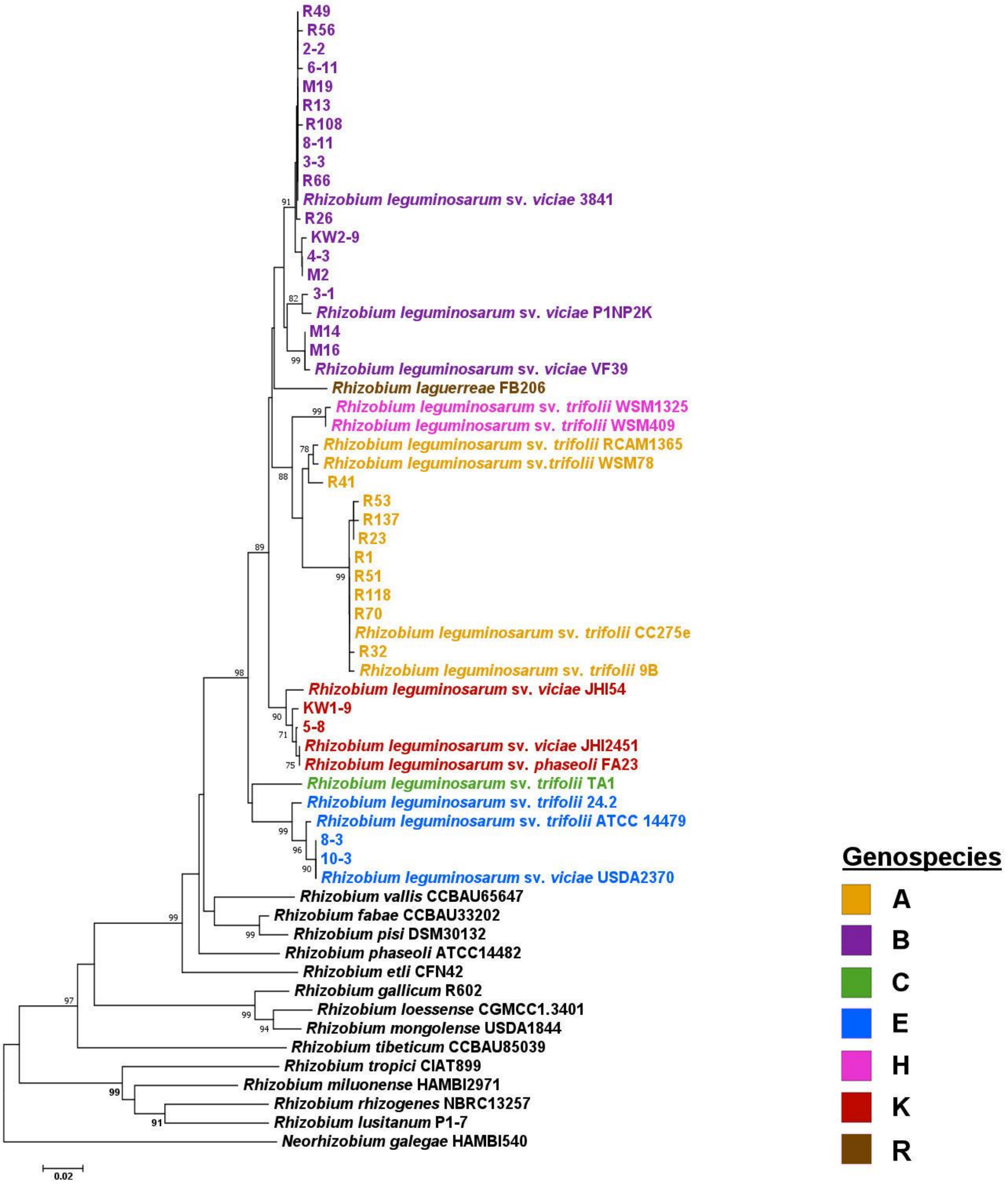

Figure S8: Phylogenetic tree based on the *glnII* sequence (618 bp) showing relationships of the representative red clover isolates with selected reference strains for different rhizobial species. Bootstrap values are shown on the branches. The scale bars represent nucleotide substitutions. Colors indicate genospecies.

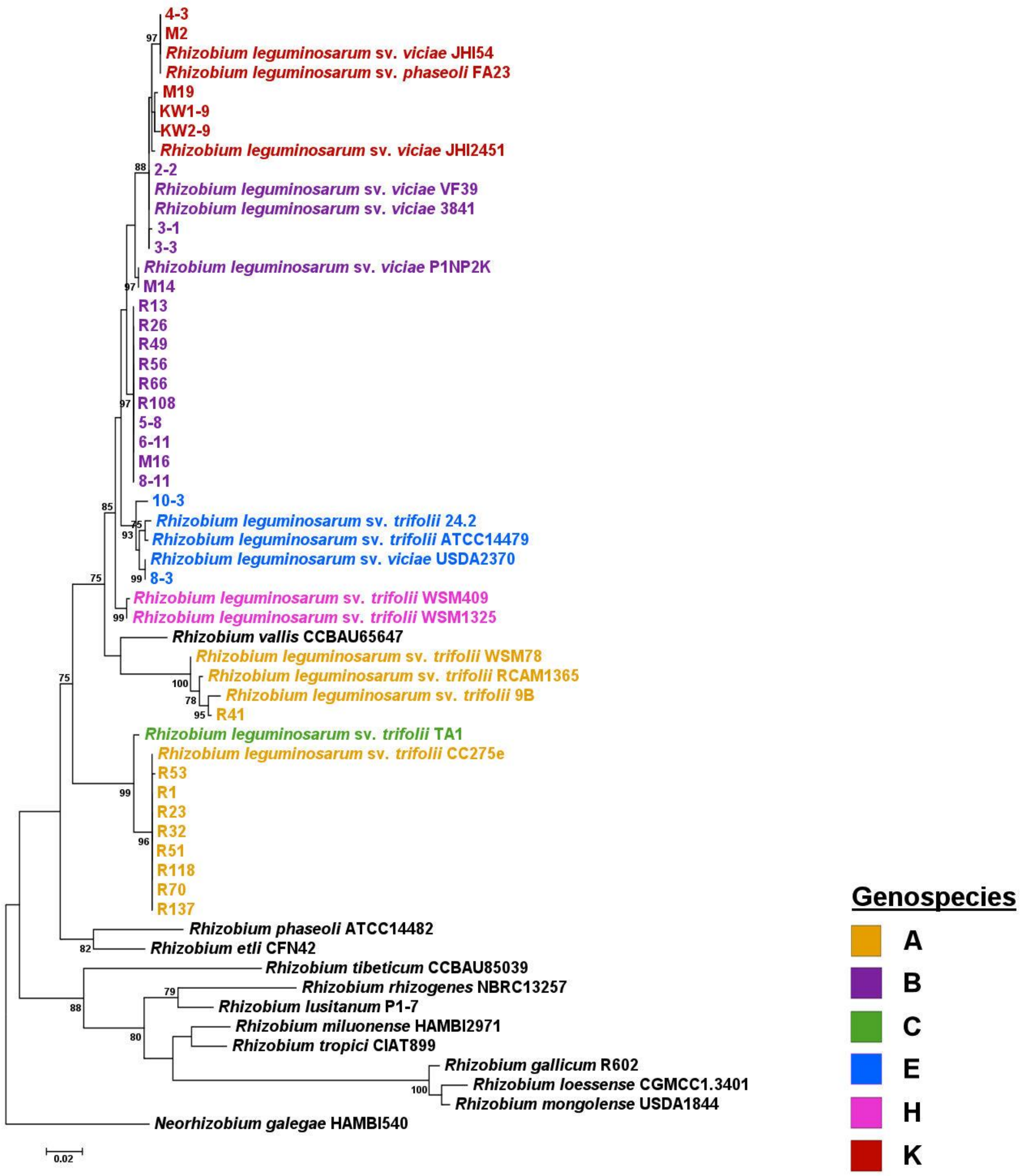

Supplement: Supplementary file 1 — Supplementary Information. [file 41598_2022_16410_MOESM1_ESM.pdf]
